# Predictive correlates of poor sleep associated with increased risk of severe asthma exacerbations among children with moderate‐to‐severe asthma

**DOI:** 10.1111/pai.70229

**Published:** 2025-10-24

**Authors:** Anuja Bandyopadhyay, Bowen Jiang, Yash Shah, Arthur H. Owora

**Affiliations:** ^1^ Division of Pediatric Pulmonology, Allergy/Immunology and Sleep Medicine, Department of Pediatrics Indiana University School of Medicine Indianapolis Indiana USA; ^2^ Center for Biomedical Informatics Regenstrief Institute Indianapolis Indiana USA

**Keywords:** electronic health records, pediatric asthma, polysomnography, prognosis, severe exacerbations, sleep disturbance, sleep latency

## Abstract

**Background:**

Sleep studies (polysomnography) are a diagnostic tool used to monitor various physiological parameters during sleep to diagnose and manage sleep disorders. However, the prognostic utility of sleep measures for the prediction of childhood asthma severe exacerbation (SE) risk is unknown.

**Methods:**

Retrospective cohort analysis to identify correlates and quantify the prognostic utility of poor sleep measures for the prediction of SE risk among children with moderate or severe asthma.

**Results:**

The study cohort included 161 patients (36% female, 33% African American, mean (standard deviation [SD]) age of 10 [4] years). A higher sleep arousal index (i.e., sleep fragmentation measured as disruptions in brainwave activity) was associated with increased risk of SE among male (adjusted odds ratio [aOR]: 1.13, 95% CI: 1.04, 1.23) but not female patients (aOR: 0.97, 95% CI: 0.88, 1.07). A history of SE(s) and use of inhaled glucocorticoid plus a long‐acting β2‐agonists (ICS plus LABA) were associated with higher odds of SE; conversely, a history of sleep latency reducing medication was associated with lower odds of SEs (*p* < .05). Inclusion of these sleep‐related factors in the multivariable model to predict SE had higher prognostic accuracy than a model based on history of SE(s) alone (*p* < .01).

**Conclusion:**

In addition to prior SE(s), elevated sleep arousal index among male children, use of ICS plus LABA, and history of untreated sleep disturbance can improve the accuracy of SE risk prognosis to inform targeted preventive interventions to reduce excess acute healthcare utilization among children with comorbid sleep problems and moderate/severe asthma.

**Abbreviations**
AASM: American Academy of Sleep Medicine
AUC: Area under curve
BMI: Body Mass Index
CDC: Center for Disease Control and Prevention
CO2: Carbon dioxide
DCA: Decision curve analysis
EHR: electronic health record
ER: Emergency Room
FEV: Forced Expiratory Volume
ICS: Inhaled corticosteroids
LABA: Long‐acting beta agonists
NREM: Non‐Rapid Eye Movement
PDM: Passive digital marker
PSG: Polysomnography
SE(s): Severe exacerbation(s)

**Key message**

Children with moderate or severe asthma and poor sleep are at increased risk of severe asthma exacerbations that result in increased acute healthcare utilization. Our study findings show that among these at‐risk children, an elevated sleep arousal index among male children, use of ICS plus LABA, and untreated sleep disturbance are associated with increased risk of severe asthma exacerbations. Moreover, these predictors improve the prognostic accuracy for identifying at‐risk children who may benefit from targeted preventive intervention to reduce the risk of future severe asthma exacerbations.

## 1 INTRODUCTION

Childhood asthma and sleep disorders are prevalent and may concurrently result in increased morbidity and acute healthcare utilization. The majority of children with uncontrolled asthma often present with complaints of insomnia, poor sleep quality, difficulty falling asleep, and sleep disruptions. On the other hand, poorly controlled asthma symptoms may impair sleep. However, there is a paucity of research on how and to what extent poor sleep markers may predict the risk of severe asthma exacerbations that result in excess acute asthma‐related healthcare utilization.

Severe asthma exacerbations are often preceded by a period of poor asthma control.^1^ Thus, there may be a window of opportunity during poor asthma control where aggressive therapy that addresses contributory comorbidities such as sleep problems may prevent future severe exacerbations. However, predicting severe childhood asthma exacerbations is challenging because of the complex interaction of risk factors such as patient characteristics (race/ethnicity, age, sex, family history of asthma, allergies, eczema), adherence issues, environmental triggers, and comorbidities that may vary across children with moderate or severe asthma.^2^ Therefore, it is difficult to identify any single factor or a one‐size‐fits‐all prediction model that can reliably and accurately predict severe exacerbation risk among all children with moderate or severe asthma.^3^ Consequently, the initiation of preventive interventions or treatment is often delayed, resulting in preventable severe exacerbations and associated excess acute healthcare utilization.

Although sleep studies are routinely performed in patients with asthma,^4^ no studies to our knowledge have examined whether physiological signals measured in these studies can be leveraged to identify patients at risk of severe exacerbations. Therefore, the objective of our study is two‐fold: identify correlates of poor sleep that are associated with increased risk of severe exacerbations 12 months post a sleep study and quantify the prognostic utility of sleep measures for predicting risk of severe exacerbations among children with moderate or severe asthma. We hypothesize that poor sleep measures such as sleep fragmentation (higher arousal index), reduced sleep (lower sleep efficiency), decreased oxygenation (mean spO2), hypoventilation (CO2), and sleep apnea (higher apnea hypopnea index) are associated with increased risk of severe exacerbations. In addition, including these factors within a multivariable model framework can improve the prognostic accuracy of severe asthma exacerbation risk.

## 2 METHODS

### 2.1 Study design and data sources

**FIGURE 1:**
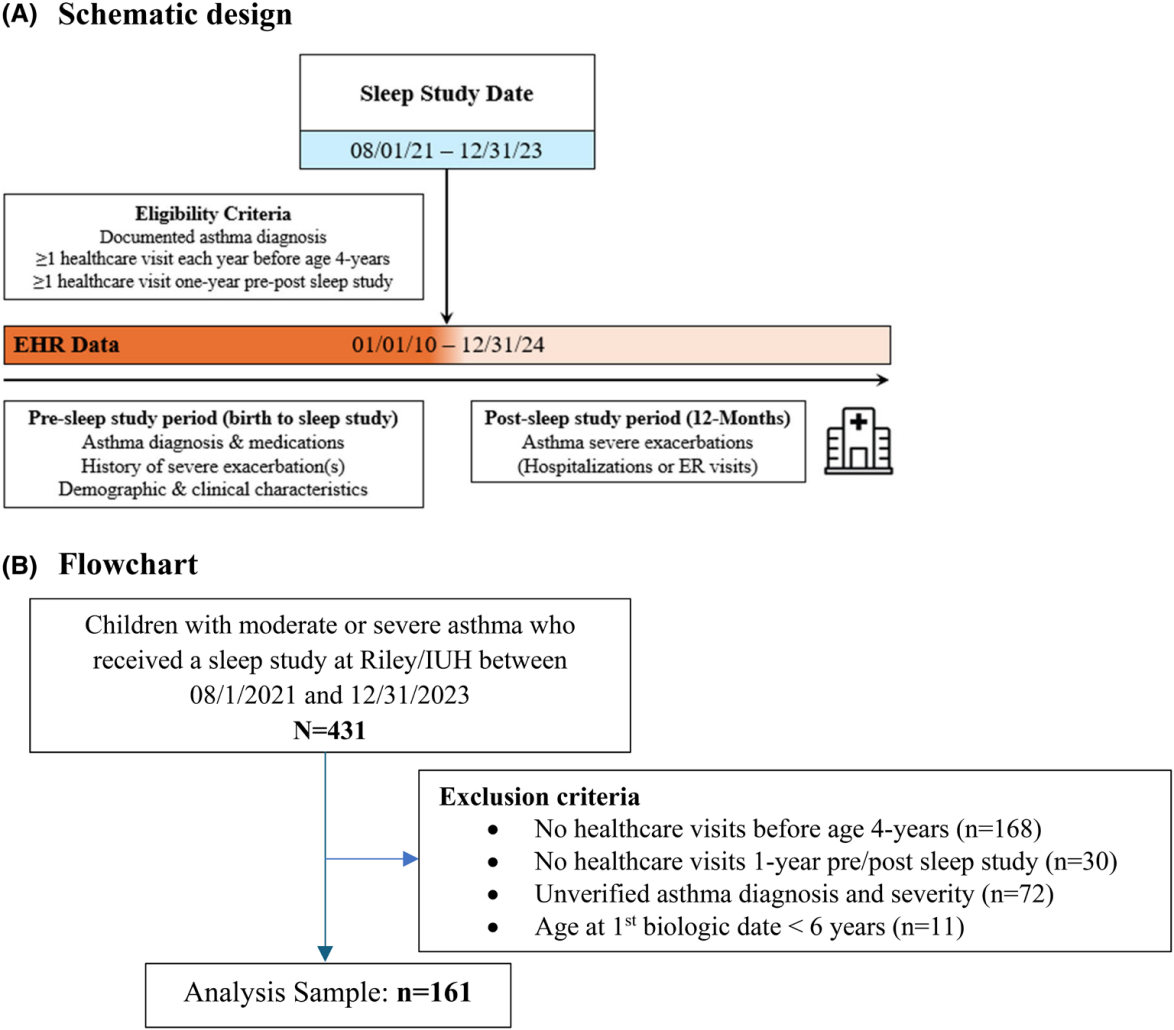
Study design schematic and flow chart. Encounter data accrued between January 1st, 2010 and December 31st, 2024, were examined to allow for a reasonable duration of patient follow‐up period before and after the sleep study. The longer pre (vs. post) sleep study period was considered to ensure sufficient data was available to characterize early‐childhood asthma risk factors (≤3 years of age), history of disease severity, and prior treatments.

We conducted a retrospective cohort study in a real‐world setting that included children diagnosed with moderate or severe asthma who had a sleep study between August 2021 and December 2023 (Figure 1). Sleep measures were obtained from the pediatric sleep program databases. Demographic and patient clinical/medication history were retrospectively generated from the electronic health record (EHR) data warehouse at Indiana University Health (IUH) system affiliated institutions. IUH is a comprehensive healthcare system that provides primary and subspecialty care for over 1.2 million residents in Indiana. In addition, Indiana Network for Patient Care (INPC) EHR databases that encompass over two‐thirds of the healthcare institutions in Indiana (including IUH) were used to obtain data on patient diagnoses, medication orders, and prescriptions from encounter‐specific records (inpatient, outpatient, and ER visits); this helped minimize the likelihood of omitting asthma‐related encounters for the cohort that might have occurred outside the IUH system. This study was approved by the Institutional Review Board (IRB) at Indiana University School of Medicine, with a waiver consent for de‐identified data (Protocol #:15869).

### 2.2 Study cohort

Eligible patients for study analyses had at least one healthcare visit before age 4 years, at least 1 year of pre/post sleep study follow‐up, and a pulmonologist‐verified asthma diagnosis and severity level. EHR data was available for 2010–2024, so we extracted data for patients who had their sleep study between 2021 and 2023, to allow at least 12 months of follow‐up after the sleep study.

### 2.3 Primary and secondary outcome

Our primary outcome was the occurrence of at least one acute severe asthma exacerbation, defined per American Thoracic Society and the European Respiratory Society (ATS/ERS) guidelines as an exacerbation that resulted in a hospitalization or ER visit during a 12‐month follow‐up period after the sleep study.^5^ Oral corticosteroid prescriptions not associated with a hospitalization or ER were excluded from analysis to reduce the likelihood of outcome misclassification, since we could not determine from the EHR whether they were prescribed specifically for an acute asthma exacerbation. Our secondary outcome included a count of severe exacerbations 12 months post the sleep study categorized as zero (0), 1, or greater than one (≥2) to quantify recurrence risk.

### 2.4 Sleep measures

Sleep study or polysomnography (PSG) tests were attended, performed at an altitude of 732 feet above sea level, at an ambient temperature of 20°C–23°C, and lasted 8 h. PSG tests were performed in technical accordance with standards proposed by the American Academy of Sleep Medicine (AASM). Table S1 provides the definitions of the sleep measures that were scored by four board‐certified sleep medicine pediatric pulmonologists, in accordance with AASM guidelines/recommendations. There was a high level of interrater agreement between the pulmonologists' scores (kappa statistic: 0.9; *p* < .0.01).

### 2.5 Clinical and Medication History

Table S2 provides a summary of ICD9/ICD10 codes and key terms used to create clinical covariates. We calculated the burden of early‐childhood asthma risk factors using our validated passive digital marker (PDM) score^6^: As previously described, the PDM is constructed using EHR data at ≤3 years (a period of diagnostic uncertainty for asthma) and includes sex, race, history of parental asthma, eczema, wheezing with or without a cold, pneumonia, bronchiolitis, croup, and allergic sensitization and/or allergy reports.^6^ Patient medication history included age at first prescription for inhaled corticosteroids (ICS), ICS plus long‐term acting beta‐agonist (ICS + LABA), or oral corticosteroids and commonly prescribed sleep latency–reducing medications (hydroxyzine, melatonin, clonidine, and trazodone).

### 2.6 Statistical analysis

Descriptive statistics were used to summarize patient demographic and clinical/medication history by the occurrence of at least one severe exacerbation 12 months post the sleep study. We used Kaplan–Meier curves to characterize the time from a patient's sleep study date to an incident severe asthma exacerbation.

We examined the association between sleep measures, demographic factors, clinical/medication history, and the severe exacerbations as both a dichotomous and a possibly censored outcome (i.e., time from sleep study to severe exacerbation) using logistic and Cox proportional hazards regression models, respectively. For our logistic models, the outcome variable was the occurrence of severe exacerbation within 12 months of the sleep study (yes/no). Multivariable (binomial and multinomial) logistic regression models were used to identify factors associated with an increased risk and higher frequency of severe exacerbations 12 months post‐sleep study. For our Cox model, the outcome variable was the time to a severe exacerbation starting on the date of the sleep study until a patient's first severe exacerbation or the end of study follow‐up (12/31/2024), whichever comes first. Patients were right censored if the study end date was reached before the occurrence of a severe exacerbation. We examined pairwise interaction terms to test whether the relationship between sleep measures and the risk of severe exacerbation differed by a patient's demographic factors or clinical/medication history. In the absence of modified effects (i.e., statistically significant interaction terms), multivariable models were used to determine to what extent sleep measures were associated with increased (or earlier) odds of severe exacerbations adjusted for potential confounders in the final models.

#### 2.6.1 Model selection and estimation for predicting severe exacerbations

Prediction features (PFs)/predictors were selected based on statistical and clinical criteria. We used multivariable logistic regression with a least absolute shrinkage and selection operator (LASSO) method for the selection of model predictors.^7^ The optimal penalty for predictor selection determined based on a 10‐fold cross‐validation was 0.08. After LASSO selection, additional clinically relevant predictors (sleep measures, demographic factors, clinical/medication history) were manually added and retained in the final model if they resulted in improved prognostic accuracy (i.e., an increase of Area Under the Curve [AUC] by at least 0.01). We reduced the LASSO penalty to 0.05 (rather than 0.08) to retain sex and asthma‐related medication history as clinically relevant variables in our final prediction model. The final PFs met the minimum recommendation of 10 events (i.e., severe exacerbations) per variable needed to minimize overfitting and optimism during the development of a logistic regression model.^8^ The final model was evaluated by constructing a receiver‐operating characteristic (ROC) curve using a linear predictor. The linear predictor was calculated by summing up each LASSO penalized β‐coefficient multiplied by the corresponding variables' values. Youden's index was used to select a cutoff score that maximized sensitivity and specificity.

#### 2.6.2 Model internal validation

Model prognostic performance was internally validated using a 10‐fold cross‐validation routine for each performance metric (e.g., AUC, calibration intercept, calibration slope, and measures of clinical utility) to estimate bias‐corrected estimates.^9^

#### 2.6.3 Clinical utility

Decision curve analysis (DCA)^10^ was used to evaluate the ability of our model to provide information that leads to improved patient care and health outcomes. DCA evaluated the potential benefits and harms of using our model across a range of threshold probabilities, which represent a clinician's willingness to act on a predicted risk. By plotting net benefit against predicted risk thresholds, DCA helps determine if a model improves clinical decision‐making compared to standard approaches like “treat all” or “treat none”. Improvements in prognostic accuracy (vs. using history of severe exacerbations – a proxy for current practice) for risk prediction were summarized using a continuous net reclassification improvement index and net benefit.^10^ We also constructed a nomogram to provide a visual representation of our prediction model. Kaplan–Meier analysis was used to quantify the potential impact of risk classification after the sleep study for earlier detection of patients at risk of severe exacerbations.

Two‐tailed *p*‐values <.05 were considered statistically significant. All data analyses were conducted using R version 4.4.3. We followed the Strengthening the Reporting of Observational Studies in Epidemiology reporting guidelines for cohort studies and Transparent Reporting of a Multivariable Prediction Model for Individual Prognosis or Diagnosis guidelines.^11^

## 3 RESULTS

### 3.1 Study participants

Our study cohort included 161 children with moderate or severe asthma who received healthcare from IUH and completed a sleep study between August 2021 and December 2023 (Figure 1). Table 1 summarizes the patient characteristics: briefly, 36% were female, 32.9% were Black, and 11.8% were Hispanic/Latino. Cohort patients had an average (standard deviation) age of 5 (4) years at their incident asthma diagnosis and 10 (4) years when they completed the sleep study. At the time of the sleep study, the majority (77.6%) of the patients were overweight or obese. Overall, study patients had adequate pulmonary function with an average (SD) Forced Expiratory Volume in 1 s (FEV1) predicted percent of 102 (12). Based on the PDM score constructed using risk factors at ≤3 years of age, 53.4% of patients had a moderate‐to‐high burden of early childhood asthma risk factors. In the year preceding the sleep study, 42.9% of the patients had at least one severe exacerbation, 28.6% had at least one ICS plus LABA prescription, and only 19.3% had at least one commonly prescribed sleep latency reducing medication. The distribution of specific sleep latency reducing medication prescription history is summarized in Table S3. All patients had at least 1 year of follow‐up after the sleep study.

**TABLE 1:** Twelve‐month cumulative incidence of severe asthma exacerbations (SE) and associated demographic and clinical correlates post‐sleep study.

Patient characteristics	Overall *N* (%)	Incidence SE *n* (%)	*p* Value	Odds ratio (95% CI)	
				Crude	Adjusted
Overall	**161**	52 (32.3%)			
Sex					
Female	58 (36.0%)	16 (27.6%)	.337	Ref	Ref
Male	103 (64.0%)	36 (35.0%)		1.34 (0.66, 2.77)	1.41 (0.67, 3.08)
Race					
White	101 (62.7%)	37 (36.6%)	.112	Ref	Ref
Black	53 (32.9%)	15 (28.3%)		0.68 (0.33, 1.39)	0.57 (0.25, 1.25)
Others^c^	7 (4.3%)	0 (0.0%)		–	–
Ethnicity					
Non‐Hispanic/Latino	142 (88.2%)	45 (31.7%)	.652	Ref	Ref
Hispanic/Latino	19 (11.8%)	7 (36.8%)		1.43 (0.43, 3.98)	1.65 (0.52, 5.03)
Age (years)					
Asthma Diagnosis Mean (SD)	5 (4)	4 (4)	.**023**	0.92 (0.85, 1.00)	1.03 (0.90, 1.18)
Sleep Study Mean (SD)	10 (4)	10 (5)	.639	0.97 (0.90, 1.05)	1.02 (0.93, 1.12)
SE History^a^					
0	92 (57.1%)	18 (19.6%)	**<.001**	Ref	Ref
1	29 (18.0%)	11 (37.9%)		2.33 (0.91, 5.75)	1.89 (0.73, 4.83)
>1	40 (24.8%)	23 (57.5%)		**5.43 (2.42, 12.6)**	**3.88 (1.58, 9.77)**
PDM Risk					
Low	75 (46.6%)	18 (24.0%)	.**054**	Ref	Ref
Moderate	47 (29.2%)	16 (34.0%)		1.51 (0.67, 3.41)	0.95 (0.36, 2.39)
High	39 (24.2%)	18 (46.2%)		**2.43 (1.06, 5.62)**	1.56 (0.62, 3.91)
CDC based BMI Category					
Underweight	8 (5.0%)	2 (25.0%)	.782	Ref	Ref
Healthy Weight	28 (17.4%)	10 (35.7%)		1.76 (0.33, 13.6)	2.74 (0.43, 24.3)
Overweight	35 (21.7%)	9 (25.7%)		1.17 (0.22, 9.03)	1.35 (0.20, 12.0)
Obese	90 (55.9%)	31 (34.4%)		1.66 (0.36, 11.8)	3.60 (0.53, 33.4)
Medication History					
ICS^a^	96 (59.6%)	34 (35.4%)	.304	1.38 (0.69, 2.79)	1.20 (0.57, 2.57)
ICS plus LABA^a^	46 (28.6%)	24 (52.2%)	**<.001**	**3.51 (1.70, 7.39)**	**2.55 (1.17, 5.59)**
Sleep latency Reducing Medications^b^	66 (41.0%)	16 (24.2%)	.068	**0.48 (0.23, 0.96)**	**0.44 (0.19, 0.98)**

*Note*: Bold values have significance (*p* < 0.05.

Abbreviations: BMI, Body Mass Index; CDC, Center for Disease Control and Prevention; ICS plus LABA, long‐term acting beta‐agonist; PDM, Passive Digital Marker.

^a^ History of severe exacerbations (SE) and asthma‐related medications are determined 1‐year prior to sleep study.

^b^ Any history of sleep latency reducing medications (not restricted to the year before the sleep study): Hydroxyzine – 9 (5.6%), Melatonin – 20 (12.4%), Clonidine – 9 (5.6%), Trazodone – 4 (2.5%).

^c^ Others includes Asian (3) and Multiracial (4).

### 3.2 Cumulative incidence of severe exacerbations

**FIGURE 2:**
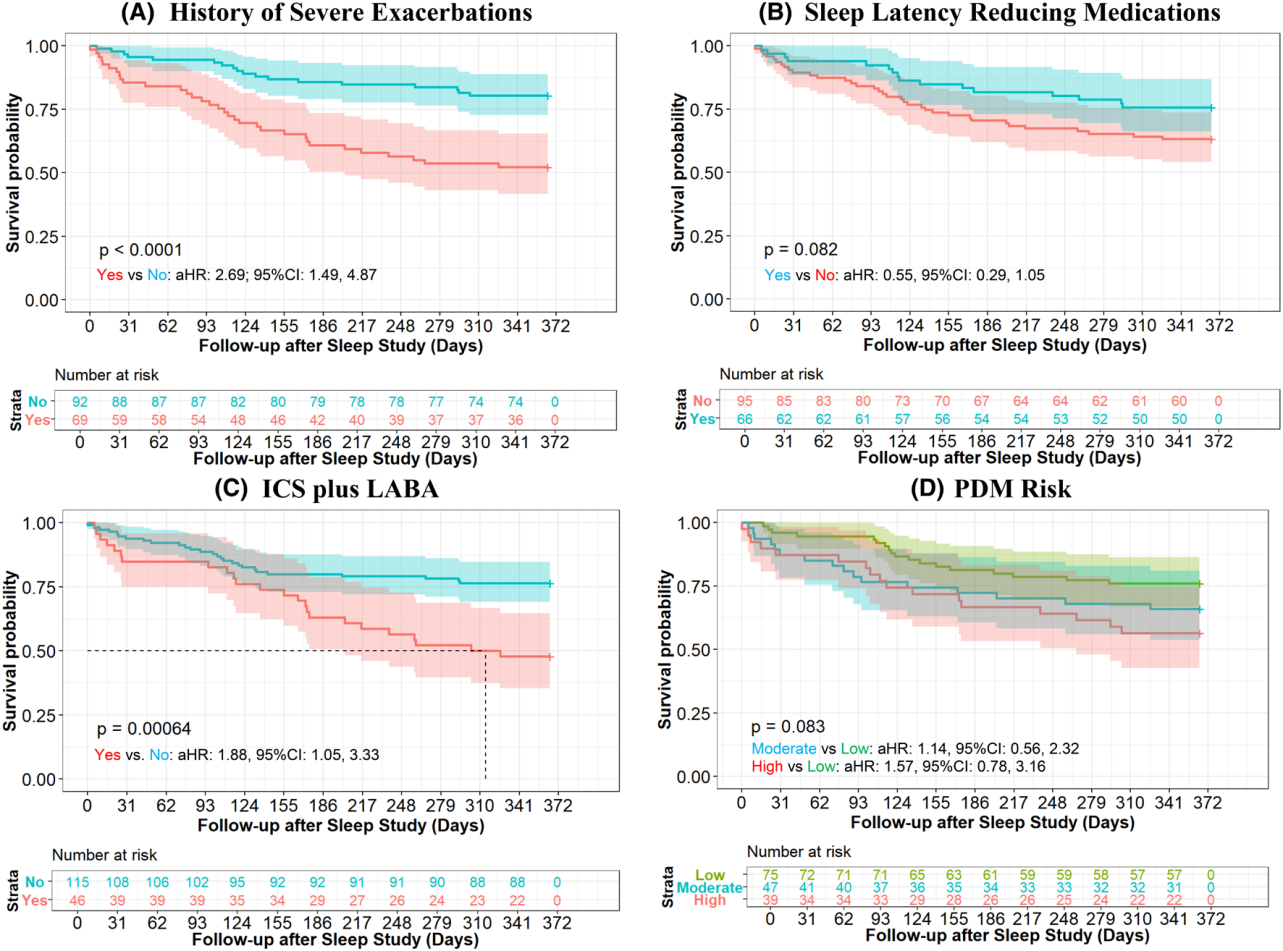
Kaplan–Meier plot of time to incident severe exacerbation 12‐months after the sleep study.

Overall, 32.3% (52) children had at least one severe asthma exacerbation during the 12‐month study follow‐up. Figure 2 shows that patients with a history of SEs (Figure 2A), no history of sleep latency reducing medications (Figure 2B), a history of ICS + LABA (Figure 2C), and higher PDM scores (Figure 2D) have earlier severe exacerbations after the sleep study.

### 3.3 Post‐sleep study correlates of severe exacerbations

**FIGURE 3:**
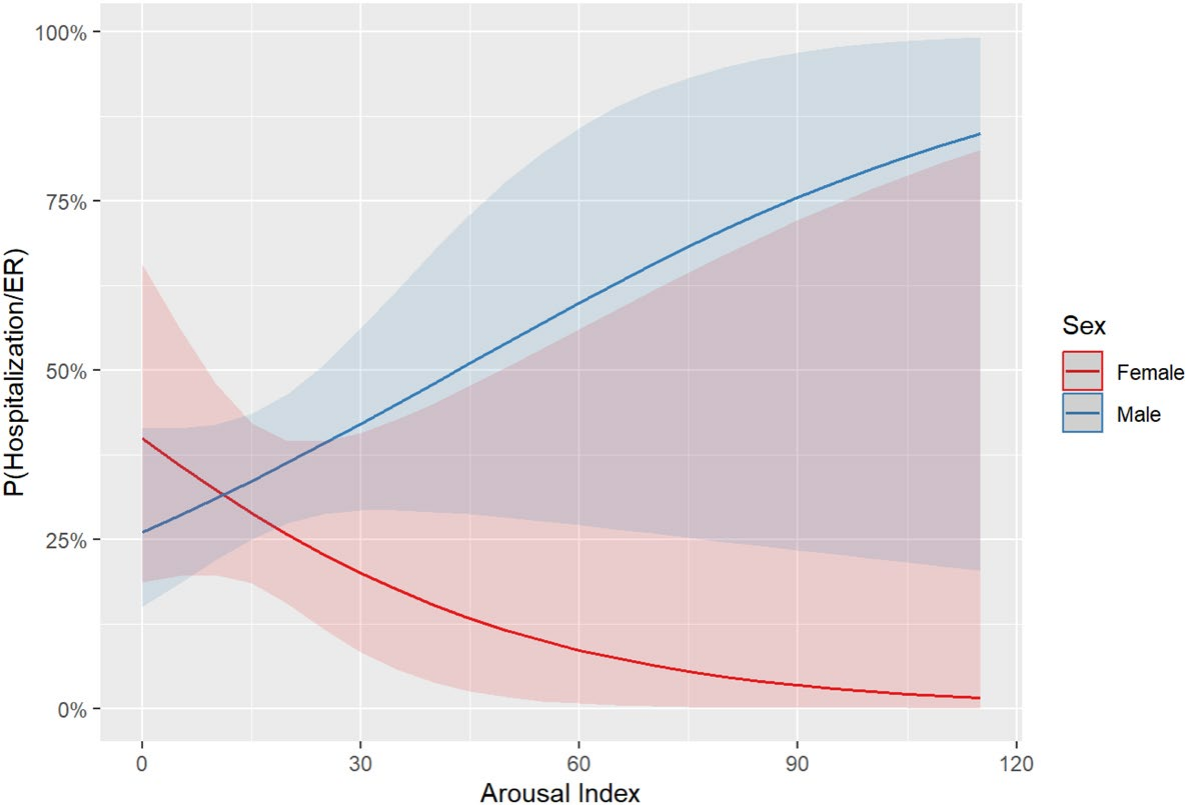
Multiple logistic regression results: The association between risk of severe exacerbations (Hospitalization/ER) and Arousal Index differs by Sex (arousal index × sex: *p* = .0118).

Multivariable logistic regression models were used to test whether poor sleep measures were associated with increased odds (binomial model) and frequency (multinomial model) of severe exacerbations. Sleep fragmentation (i.e., arousal index) was associated with increased odds of severe exacerbations among boys but not girls (sex × arousal index interaction: *p* = .0118; Figure 3); the odds of a severe exacerbation were 13% higher for every unit increase in the arousal index among boys (aOR: 1.13, 95% CI: 1.04, 1.23) but not female patients (aOR: 0.97: 95% CI: 0.88, 1.07). However, reduced sleep‐time (lower sleep efficiency), decreased oxygenation (mean spO2), hypoventilation (CO2), sleep apnea (higher apnea hypopnea index) were not associated with higher odds of severe exacerbation after adjusting for differences in patient characteristics: age, sex, and race (Table S3). Among patients who experienced one severe exacerbation post the sleep study (vs. patients who did not experience any severe exacerbations), having a history of ICS + LABA was associated with 6‐times higher odds of experiencing a severe exacerbation (Table 2 aOR: 5.86; 95% CI: 1.57, 21.9). However, having a history of ICS + LABA was not statistically associated with higher odds of more frequent (>1) severe exacerbations (Table 2 aOR: 3.46; 95% CI: 0.92, 13.0). Conversely, patients with a history of taking sleep latency reducing medications had 69% lower odds of experiencing a severe exacerbation post the sleep study (aOR: 0.31; 95% CI: 0.08, 1.24, though not statistically significant); but, these patients were 79% less likely to experience frequent (>1) severe exacerbations (aOR: 0.21; 95% CI: 0.05, 0.82, statistically significant) during follow‐up (Table 2). Children who spent a higher percentage of their Non‐Rapid Eye Movement (NREM) sleep with CO2 levels exceeding 50 mm Hg had higher odds of more frequent severe exacerbations (Table 2). A high burden of early childhood asthma risk factors (i.e., high PDM risk) was associated with higher odds of two or more severe exacerbations (Table 2). Contrary to expectation, African American (Black) Children were less likely to have more frequent severe exacerbations than Whites (Table 2). Pulmonary function, and body‐mass index at the time of the sleep study were not associated with increased risk of severe exacerbations (Table S4).

**TABLE 2:** Multinomial logistic regression results: Factors associated with one or more severe asthma exacerbations during a 12‐month follow‐up post‐sleep study.

Risk factors	One severe exacerbation			Two or more severe exacerbations		
	aOR	(95% CI)	*p*‐Value	aOR	(95% CI)	*p*‐Value
Race						
White	Ref	Ref		Ref	Ref	
Black	**0.25**	**0.07, 0.93**	.**039**	**0.25**	**0.07, 0.93**	.**039**
Average Saturations	0.75	0.46, 1.24	.267	0.70	0.46, 1.06	.094
AHI	0.94	0.86, 1.02	.125	0.93	0.86, 1.01	.082
% Total NREM sleep time with CO2 >50 mm Hg	**1.06**	**1.00, 1.12**	.**044**	**1.06**	**1.01, 1.12**	.**017**
History of ICS plus LABA use (1 year before sleep study)						
No	Ref	Ref		Ref	Ref	
Yes	**5.86**	**1.57, 21.9**	.**009**	3.46	0.92, 13.0	.066
History of any sleep latency reducing medications						
No	Ref	Ref		Ref	Ref	
Yes	0.31	0.08, 1.24	.098	**0.21**	**0.05, 0.82**	.**025**
PDM Risk						
Low	Ref	Ref		Ref	Ref	
Moderate	1.30	0.29, 5.87	.737	1.38	0.33, 5.81	.658
High	5.63	0.93, 34.0	.060	**6.97**	**1.22, 39.9**	.**029**

*Note*: Bold values have significance (*p* < 0.05.

Abbreviations: ICS plus LABA, long‐term acting beta‐agonist; PDM, Passive Digital Marker.

### 3.4 Prognostic utility of sleep measures

**FIGURE 4:**
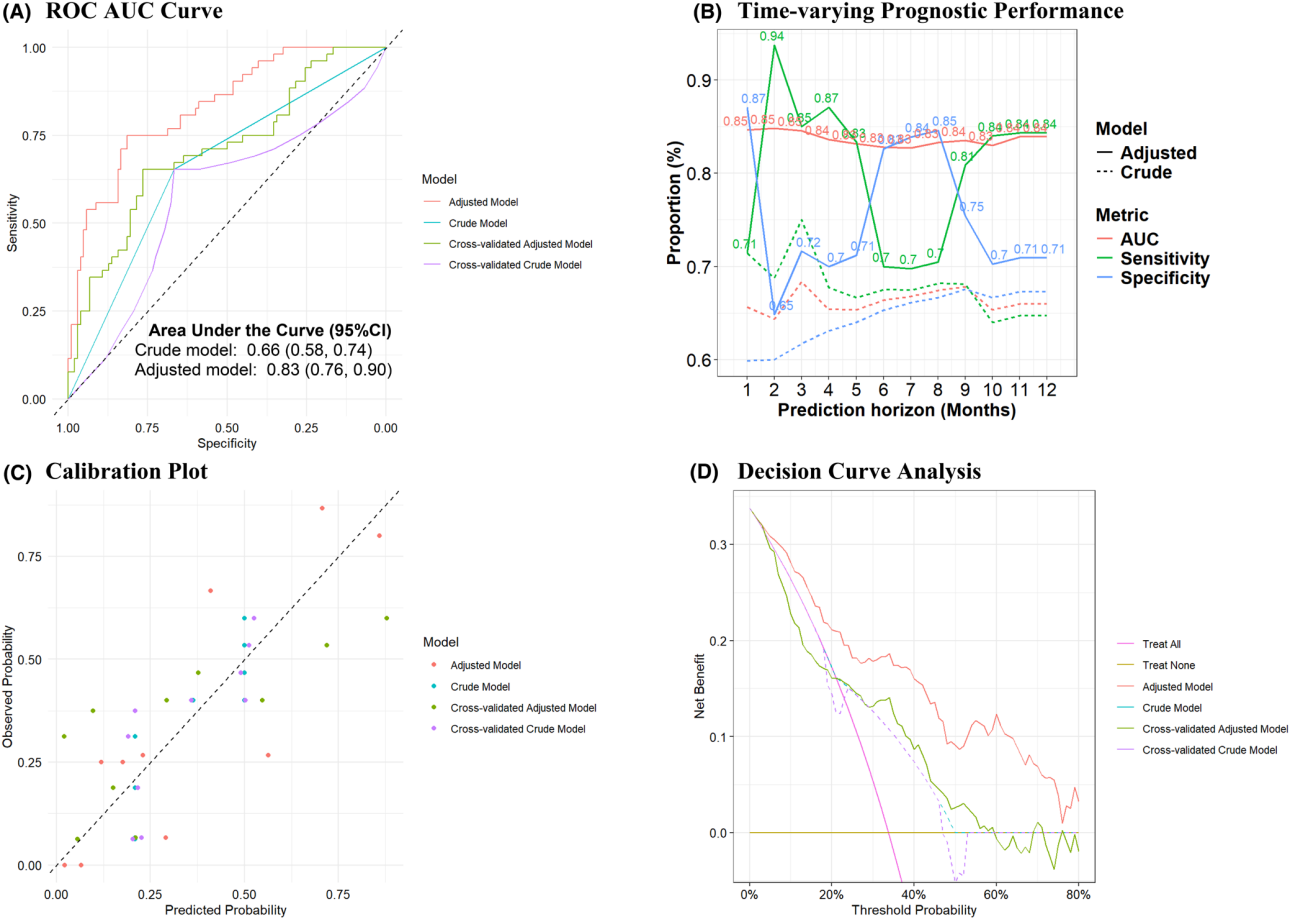
Prognostic performance of severe asthma exacerbation prediction models (corrected for optimism) after the sleep study. Comparison of the AUCs between the adjusted LASSO and crude Model (A); AUC comparisons between adjusted LASSO and crude model across different prediction horizons (B); Comparison of Calibration plots between adjusted LASSO and crude model (C); Net‐benefit comparisons across different risk threshold between the adjusted LASSO and crude model (D).

Compared to a crude model based on prior severe exacerbations (a proxy for current practice), the multivariable model including selected sleep measures (Figure 4; Figures S1–S4) had higher discrimination (Figure 4A,B), calibration (Figure 4C), and net benefit (Figure 4D) for the prediction of severe exacerbations. DCA showed that including selected sleep measures was clinically useful for risk thresholds over 21% (Figure 4D) adjusted for model optimism. Compared to the crude model, the adjusted model had a continuous (0.79; 95% CI: 0.49, 1.00) and categorical (0.49; 95% CI: 0.30, 0.67) net reclassification index that demonstrated improved discriminative performance. Similar results were observed with a reduced number of predictors in a model with less optimism (Figures S5–S8) but with lower continuous (0.43; 95% CI: 0.10, 0.75) and categorical (0.18; 95% CI: 0, 0.35) net reclassification. Risk score stratified analyses showed that the odds of severe exacerbations occurred earlier among children classified in the high (vs. low) risk strata by our prediction models (Figure S9); higher hazards were observed among patients classified as high risk compared to the use of the history of severe exacerbations (Figure 2A). Figure S10 shows that our prognostic model had stable accuracy (AUC: 0.83–0.85) with a prediction horizon (i.e., how far into the future the model can forecast severe exacerbations) of 1–12 months after the sleep study.

## 4 DISCUSSION

Our analysis shows important real‐life associations between measures of poor sleep and the risk of severe asthma exacerbations necessitating ER visits or hospitalization in children with moderate/severe asthma. Moreover, we show that including routinely collected measures of sleep quality as part of a multivariable prediction model can improve prognostic accuracy among children with comorbid asthma and sleep disorders beyond the use of the history of severe exacerbations – a proxy of current practice. To our knowledge, this is the first study to examine real‐world associations between measures of poor sleep and the risk of severe childhood asthma exacerbations. This is also the first study to evaluate the prognostic utility of sleep measures to identify at‐risk children who can be targeted for early preventive intervention to reduce the rate of excess acute healthcare utilization for asthma management.

Our study findings show that sleep fragmentation in children with adequate sleep efficiency may be a more accurate predictor of worsening asthma symptoms compared to most parameters monitoring sleep‐disordered breathing or gas exchange disturbances. A higher arousal index among boys but not girls was associated with increased odds of severe exacerbations. During sleep, male patients tend to have a stronger ventilatory response to arousals, while female patients tend to maintain better hypoxic response. Potential explanations for this sex disparity include increased production of female sex hormones leading to changes in bronchial hyperresponsiveness and atopy.^12^, ^13^ Dysynapsis, or smaller airways relative to lung size, is the proposed etiology for the male predominance in early childhood asthma hospitalizations.^14^, ^15^ Sex differences in exposure to indoor or outdoor allergens, diet, or prevalence of obesity may also explain sex disparities in asthma severity.^16^ Sleep and the immune system have a bidirectional relationship; immune activation can alter sleep, while sleep affects innate and adaptive immunity.^17^ Prolonged sleep deficiency is associated with higher risk chronic low‐grade inflammation among female than male patients.^18^ Taken together, interpreting sex‐related differences in asthma outcomes is challenging due to the difficulty of separating the effects of sex hormones from other biological factors like lung size and age, differential environmental exposures and occupational risks, and differences in symptom perception and reporting. Still, the sex disparity in the relationship between sleep fragmentation and risk of severe exacerbations requires clinical attention and consideration for asthma care management. This is particularly important if a clinician is making a prognosis or decisions about the need for preventive intervention (e.g., initiating sleep‐related disorder treatment) to attenuate the future risk of severe exacerbations.

Consistent with prior research,^2^ our study shows that children with a higher percentage of total NREM with CO2 >50 mm Hg, a previous history of severe exacerbations, and ICS plus LABA prescriptions had higher odds of severe exacerbations. The presence of hypercapnia in patients with acute severe asthma is associated with severe airflow obstruction and the need for critical care interventions.^19^ Our finding is intriguing because sleep studies were not performed during an acute exacerbation and represent baseline respiratory physiology in children with moderate or severe asthma. NREM sleep is regarded as a period of ventilatory stability during sleep when the respiratory control system maintains a stable rhythm relying on hypercapnic and hypoxic ventilatory drive. Moreover, some recent studies have suggested that high levels of CO2 can activate specific gene expressions in the lungs and can induce airway contractility.^20^ Therefore, total NREM with CO2 >50 mm Hg, as a sleep study measure, may have prognostic value as a predictor of severe exacerbation risk.

Contrary to expectation, African American (Black) children were less likely to have more frequent severe exacerbations than White children. However, self‐reported race is a social construct that is an imperfect proxy for socioeconomic status. Therefore, our finding of a lower exacerbation frequency among African American children than White children should be interpreted cautiously due to the potential effects of unmeasured confounders.

Notably, our findings also suggest a high burden of early childhood asthma risk factors (i.e., high PDM risk), independent of severe exacerbation history and sleep measures, is associated with higher odds of more frequent severe exacerbations. This finding highlights the potential utility of the burden of early childhood asthma risk factors as a predictor of severe exacerbations beyond its utility as a predictor of school‐age asthma risk.^6^

Although pulmonary function and body‐mass index are known to have independent and differing influences on sleep architecture and gas exchange polysomnography parameters in children with asthma, our study findings suggest these factors might have limited prognostic utility for severe exacerbation risk among children with comorbid sleep problems and moderate or severe asthma.

The lower odds of severe exacerbations among patients with a history of treatment involving sleep latency reducing medications are intriguing. However, there is a paucity of safety and compliance data for the use of sleep latency reducing medications for the treatment of pediatric insomnia. Although melatonin may have anti‐inflammatory effects and limit mucous production, its effect on airway smooth muscles may be detrimental.^21^ Clonidine can cross the blood–brain barrier and bind with central alpha‐2 receptors, which can decrease wakefulness, promote sleep, and increase arousal threshold without affecting the airway. Trazodone is an anti‐depressant and is primarily used in children with comorbid psychiatric disorders. First‐generation antihistaminics such as hydroxyzine are used for the treatment of insomnia, anxiety, and as an anti‐allergic in children. Though promising as a preventive strategy, the protective effect of the sleep latency reducing medications on sleep fragmentation and the risk of severe asthma exacerbation in children with sleep disorders needs to be replicated in future randomized clinical trials.

Our study has several limitations that need to be considered. One, because this was a retrospective study, except for sleep study measures, our data was limited to documentation in EHR. However, we included children who were followed in our asthma clinic longitudinally and a physician reviewed all the IUH charts for accuracy. Two, this was a single center study and thus may not be generalizable to non‐academic healthcare institutions with potentially different patient referral patterns for sleep studies. Three, all sleep studies were single night studies and therefore may be biased by study participation or first night effects. However, because most studies in the literature have shown acceptable correlation between night‐to‐night variability, we suspect such study‐related biases may not substantially affect our findings.^22^ Finally, other factors known to impact the risk of severe exacerbations, such as medication compliance and other measures of sleep fragmentation such as wake‐time after sleep onset, were not examined; this may have resulted in potential prediction misspecification bias. However, we suspect that for our study population, medication compliance may be correlated with the history of severe exacerbation (a covariate in our analysis and prediction models), but the effects of residual confounding cannot be ruled out, and therefore these unexamined risk factors should be investigated in future validation studies.

There is a known bidirectional relationship between brain to immune signaling as seen in physiological and pathological alteration during sleep in patients with chronic inflammatory diseases (including asthma).^23^ Sleep disturbances (e.g., insufficient sleep, poor sleep quality, and sleep apnea) are strongly linked to worse asthma control, increased risk of severe exacerbations, and reduced quality of life.^24^ Therefore, screening children with uncontrolled asthma for underlying sleep issues for targeted preventive intervention can improve sleep quality and reduce the risk of severe asthma exacerbations. Although including sleep studies in asthma management is clinically feasible and beneficial, particularly for patients with uncontrolled asthma, widespread barriers (e.g., cost, access, patient adherence, and the complexity of integrating care) to implementation persist.^25^ Development and evaluation of cheaper and scalable strategies for the implementation of sleep studies in clinical practice are needed.

In conclusion, despite these limitations, our findings show that leveraging existing (passive) EHR and sleep measures among children with comorbid asthma and sleep disturbance within a multivariable model framework can provide an objective and quantifiable marker of a child's risk of future severe exacerbations to inform point‐of‐care clinical decision‐making. Moreover, this can help alleviate existing barriers in risk prognosis such as the time needed to review electronic charts in busy pediatric care settings. The clinical and etiologic relevance of such considerations is underscored by the higher likelihood of earlier and more accurate detection of at‐risk patients who experience more frequent severe exacerbations (vs. current practice – where the prediction of future severe exacerbations is based only on a patient's history of severe exacerbations). At‐risk patients identified using such passive prediction models can then be targeted for preventive intervention to avert both the risk and reduce the frequency of future severe exacerbations. However, the evaluation of the efficacy of sleep‐latency reducing medication, as a plausible preventive intervention strategy– identified as a protective factor in our analysis, on reducing the rate of future severe exacerbations (especially among patients classified as ‘high‐risk’ based on our multivariable prediction model) is warranted.

## AUTHOR CONTRIBUTIONS

**Anuja Bandyopadhyay:** Conceptualization; writing – original draft; writing – review and editing. **Bowen Jiang:** Formal analysis; writing – review and editing. **Yash Shah:** Formal analysis; writing – review and editing. **Arthur H. Owora:** Conceptualization; methodology; formal analysis; writing – original draft; writing – review and editing.

## FUNDING INFORMATION

This study was in part supported by National Institutes of Health, USA grants K01HL166436 (AHO) and R03HS029088 (AHO).

## CONFLICT OF INTEREST STATEMENT

None.

## PEER REVIEW

The peer review history for this article is available at https://www.webofscience.com/api/gateway/wos/peer‐review/10.1111/pai.70229.

## Supporting information


Appendix S1.
