# Supplementary material for: Predictive correlates of poor sleep associated with increased risk of severe asthma exacerbations among children with moderate‐to‐severe asthma
Source: Pediatr Allergy Immunol. 2025 Oct 24;36(10):e70229. doi: 10.1111/pai.70229 (PMC12550647; doi:10.1111/pai.70229)
Supplement: Supplementary file 1 — Appendix S1. [file PAI-36-e70229-s001.docx]

**Supplementary Files**

**Predictive correlates of poor sleep associated with increased risk of severe asthma exacerbations among children with moderate to severe asthma**

Anuja Bandyopadhyay MD, ^a^ Bowen Jiang, MS,^a^ Yash Shah, MS,^a^ Arthur H Owora, MPH PhD,^a,b^*

^a^Division of Pediatric Pulmonology, Allergy/Immunology and Sleep Medicine, Department of Pediatrics, Indiana University School of Medicine, Indiana, USA; ^b^Center for Biomedical Informatics, Regenstrief Institute, Indiana, USA

**Corresponding author:** Arthur H Owora, MPH PhD

Dept of Pediatrics, Indiana University School of Medicine

Riley Hospital for Children

705 Riley Hospital Drive, Indianapolis, IN 46202

[ahowora@iu.edu](mailto:ahowora@iu.edu), 317-274-9109

**Supplemental Figure 1. Nomogram of the adjusted multivariable prediction model**


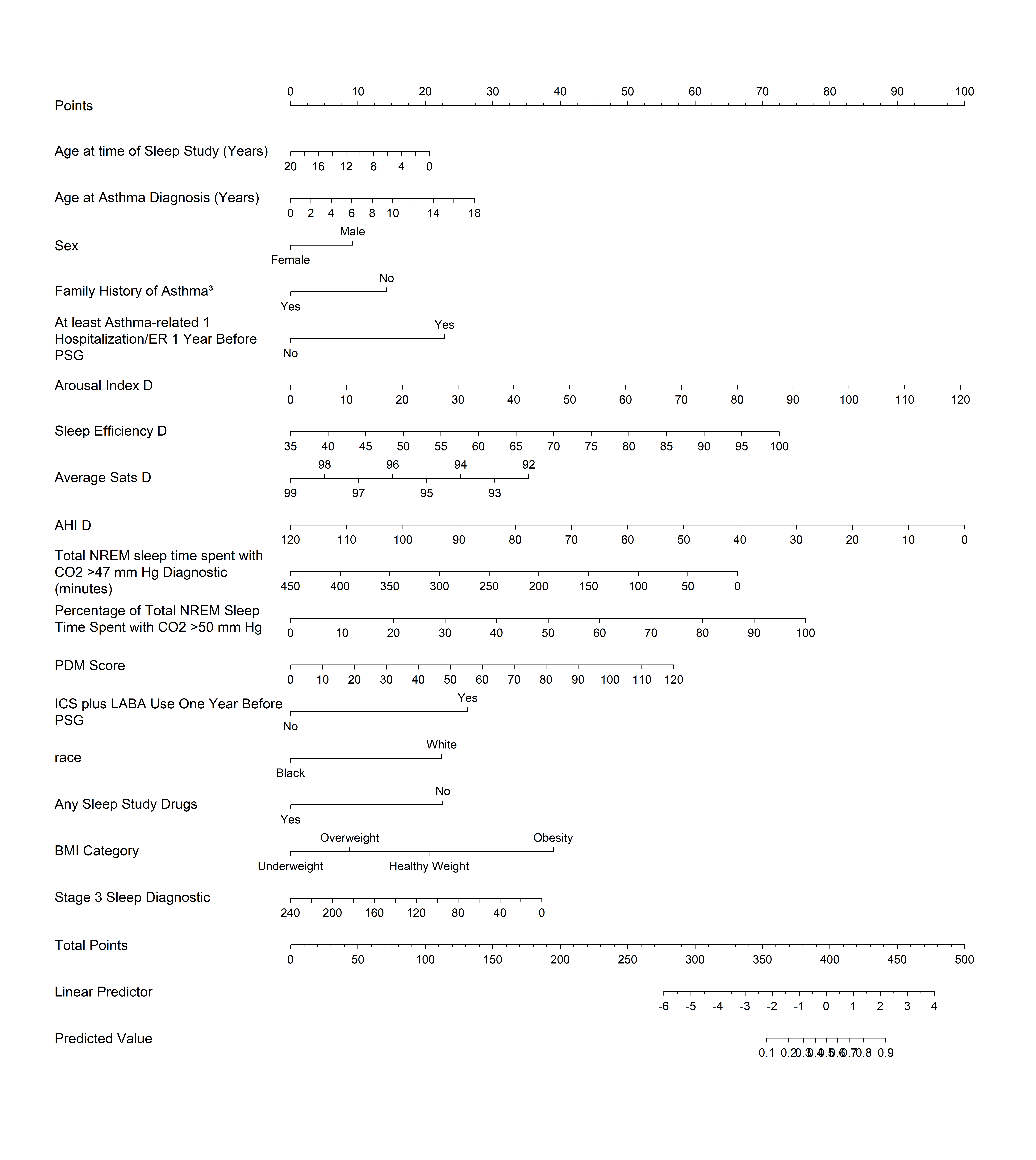


**Supplemental Figure 2.** Receiver Operating Curve Analysis of Multivariable Models with (Adjusted Model) and without (Crude Model) Selected Sleep Measures.


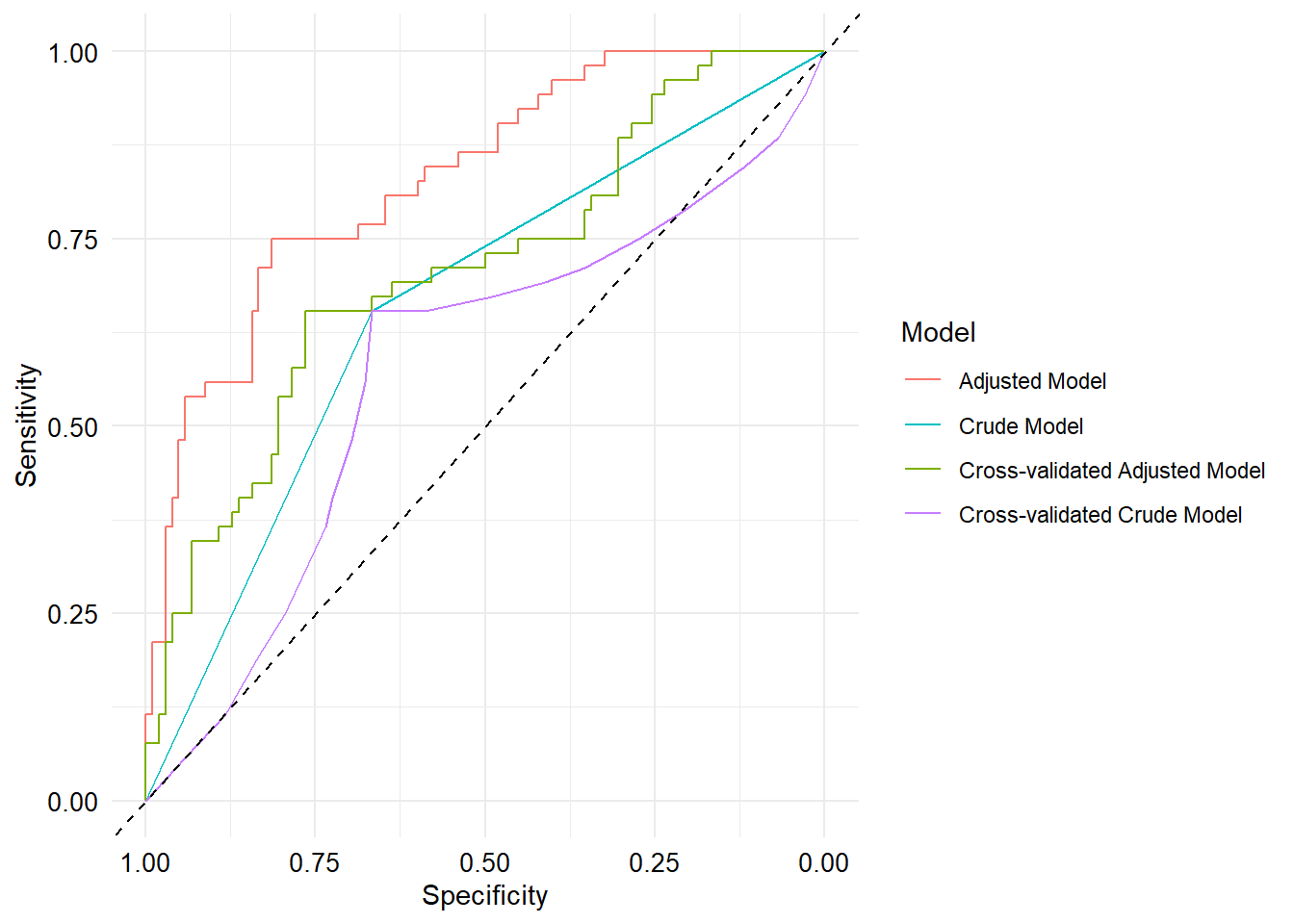


**Area Under the Curve (95%CI)**

Crude model: 0.66 (0.58, 0.74)

Adjusted model: 0.83 (0.76, 0.90)
Cross-validated adjusted model: 0.71 (0.62, 0.80)

**Supplemental Figure 3.** Calibration Plots of Multivariable Models with (Adjusted) and without (Crude) Selected Sleep Measures


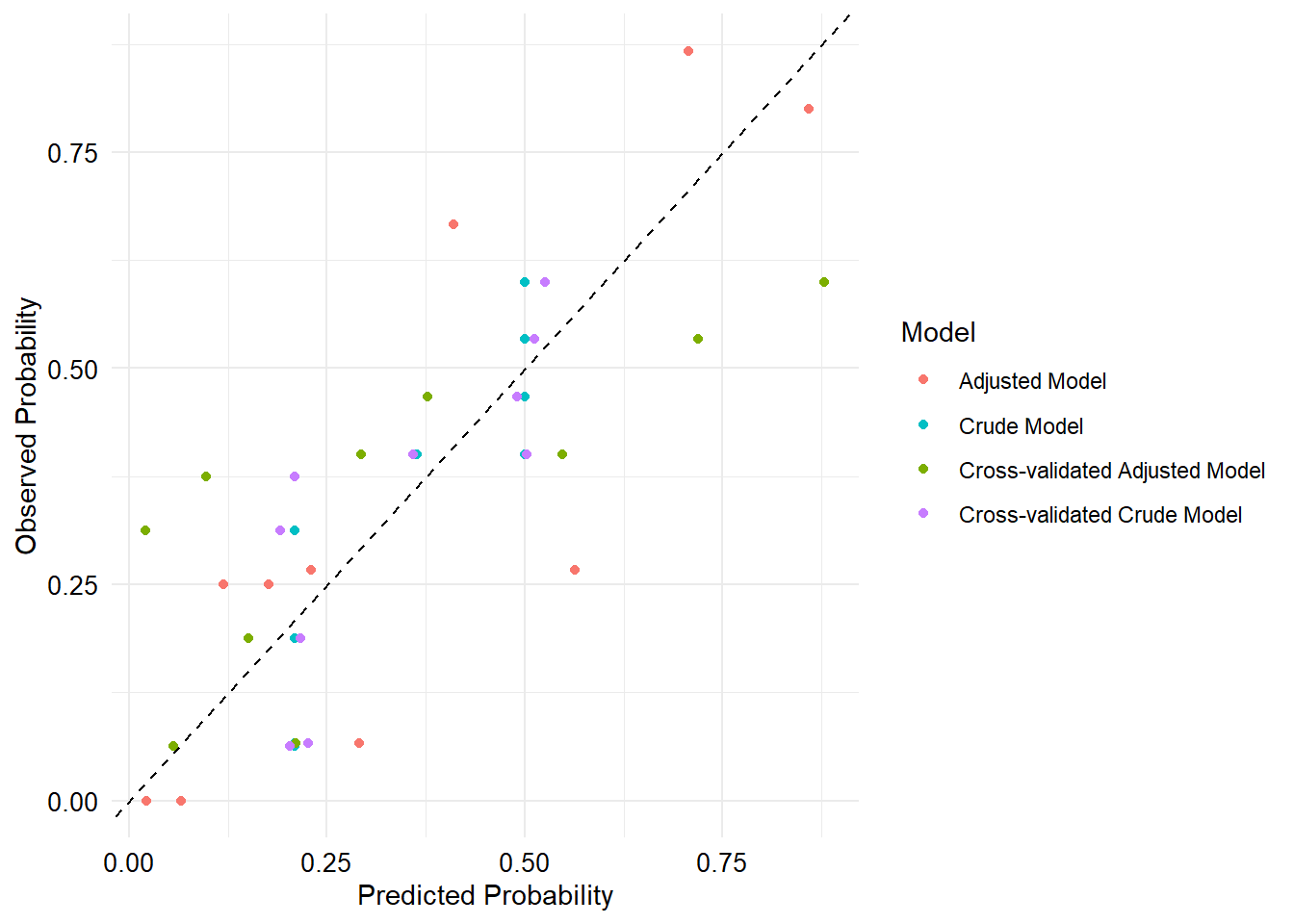


**Supplemental Figure 4.** Decision Curve Analysis of Multivariable Models with (Adjusted) and without (Crude) Selected Sleep Measures


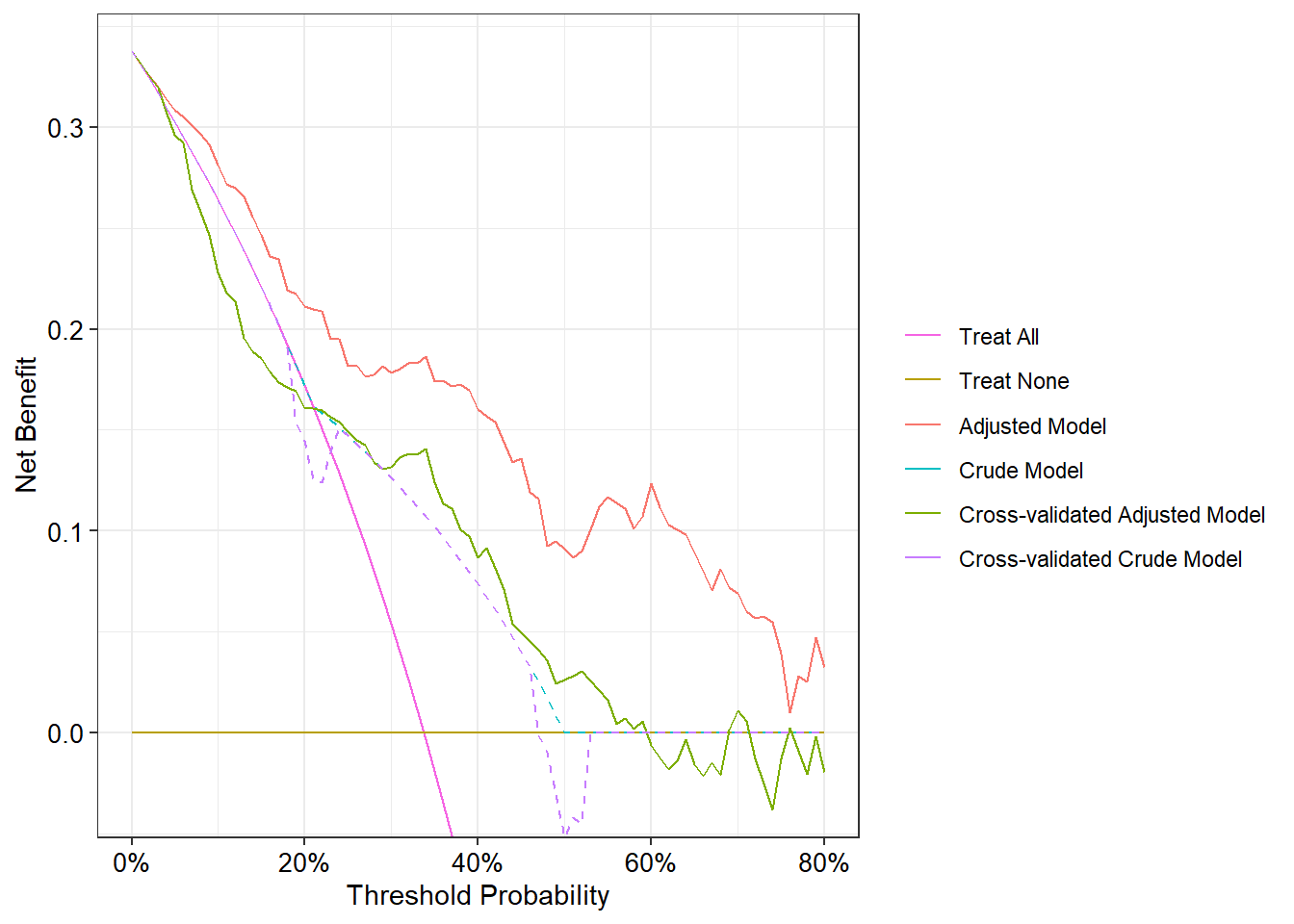


**Supplemental Figure 5. Nomogram of the simple adjusted multivariable prediction model**


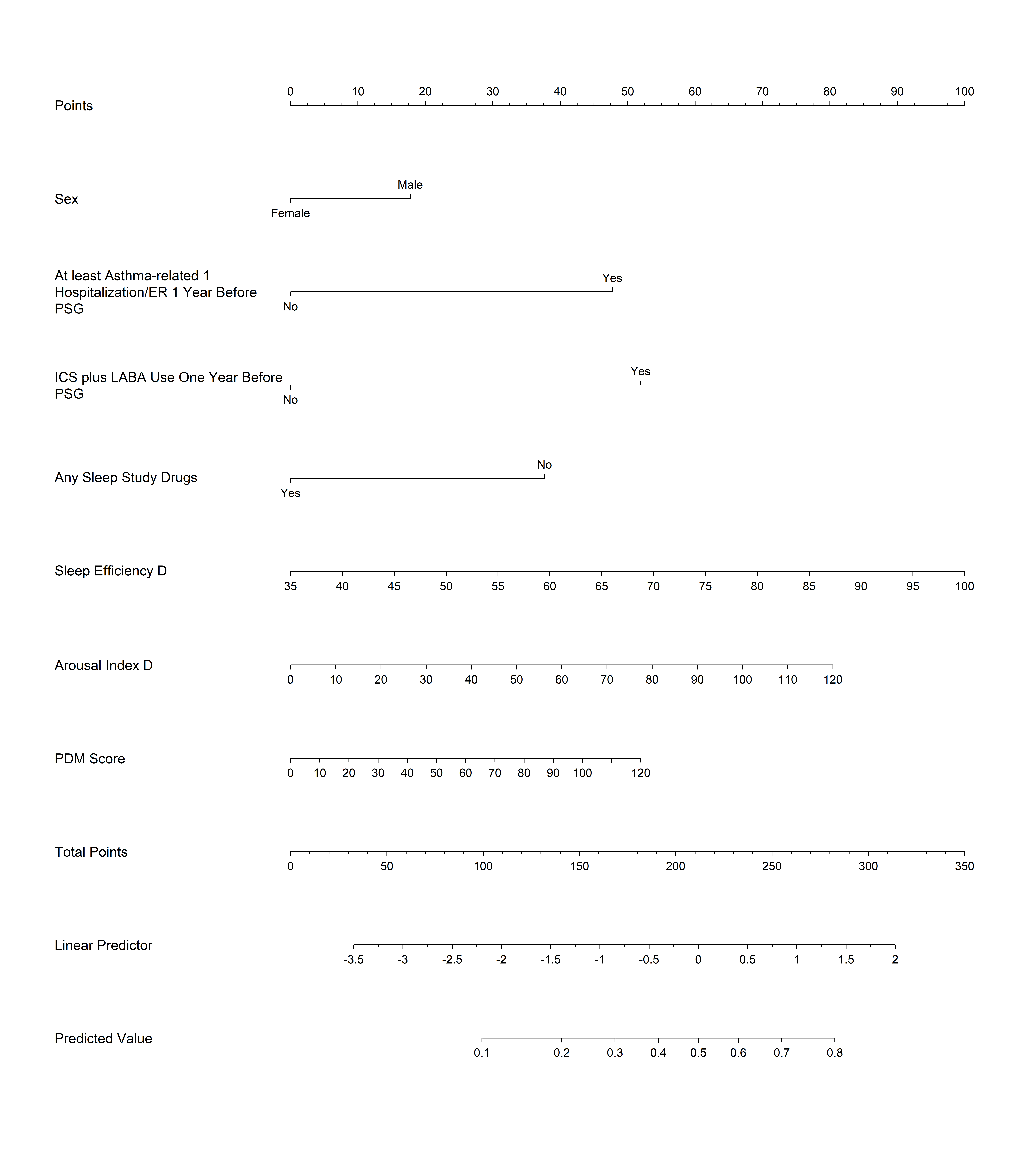


**Supplemental Figure 6.** Receiver Operating Curve Analysis of Models with (Simple Adjusted) and without (Crude) Selected Sleep Measures.


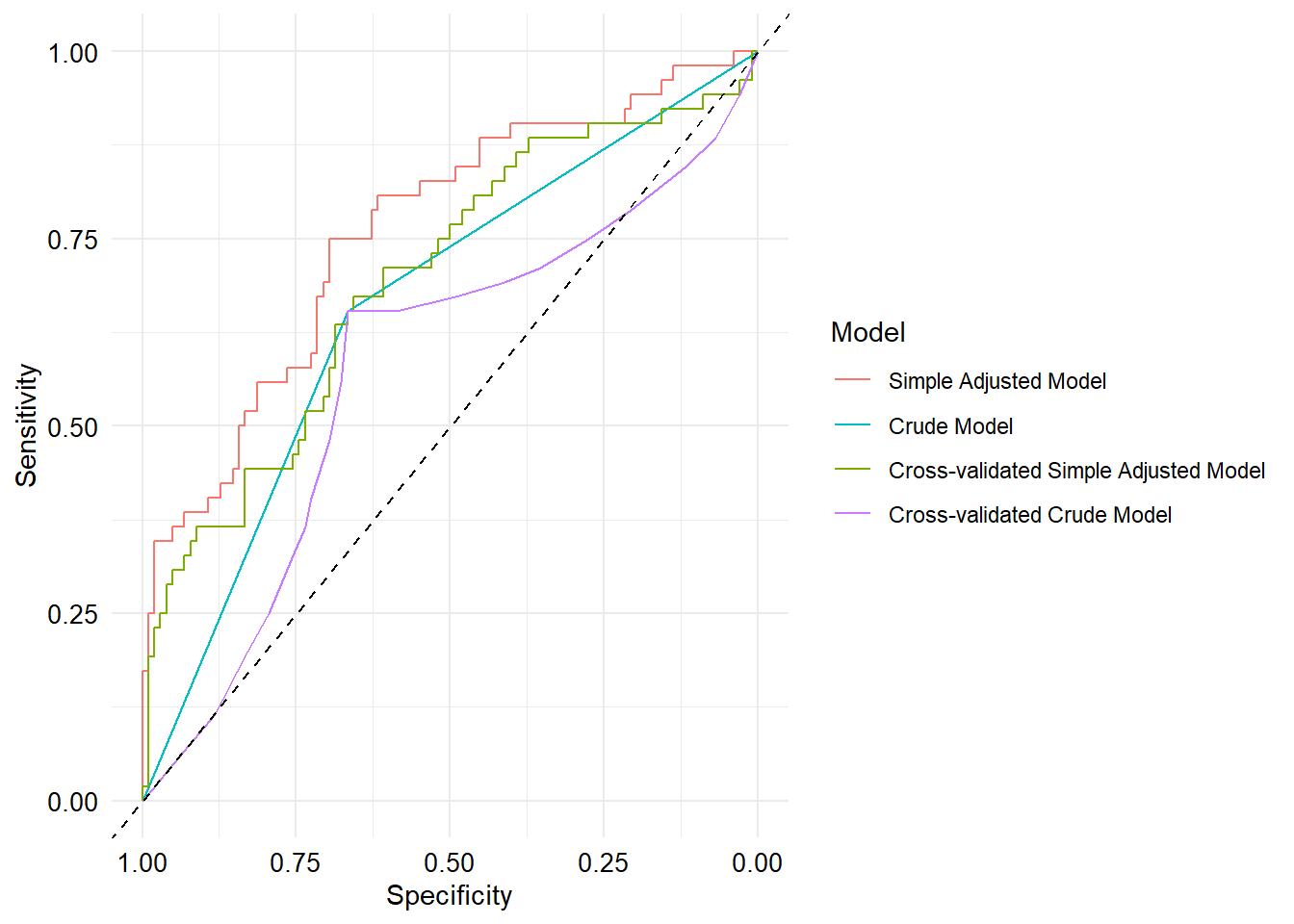


**Supplemental Figure 7.** Calibration Plots of the Multivariable Model with (Simple Adjusted) and without (Crude) Selected Sleep Measures


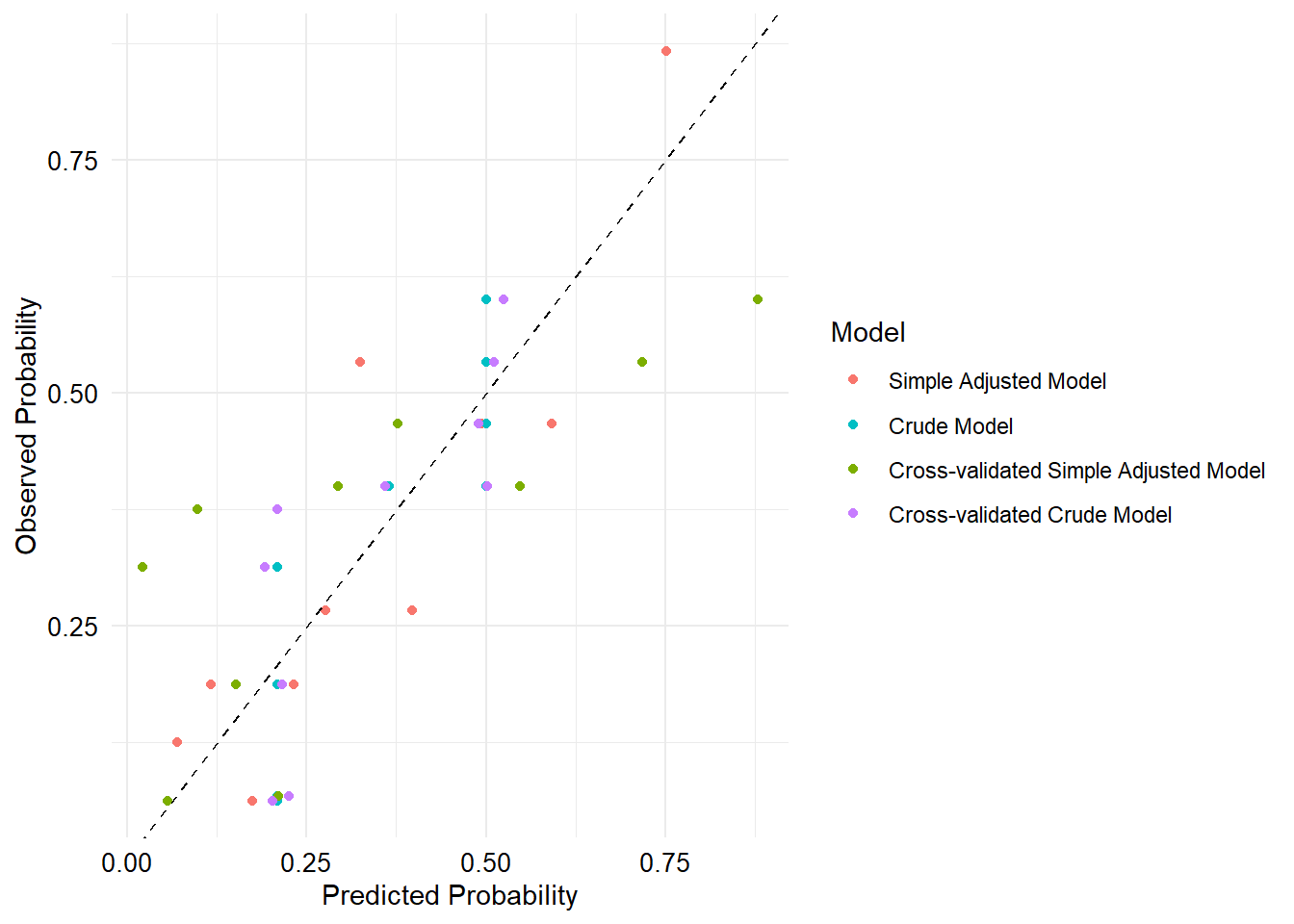


**Supplemental Figure 8.** Decision Curve Analysis of the Multivariable Models with (Simple Adjusted) and without (Crude) Selected Sleep Measures


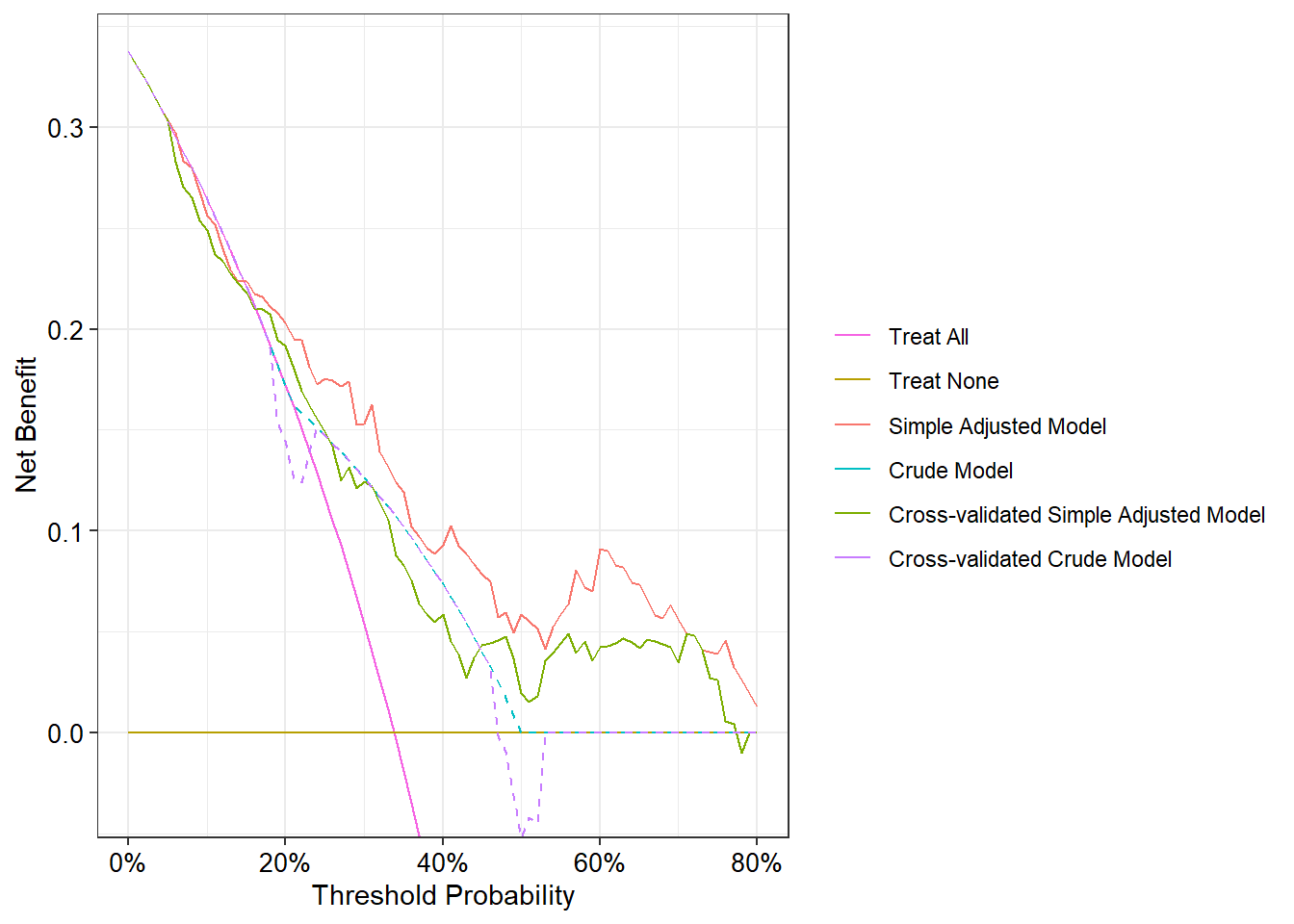


**Supplementary Figure 9. Kaplan-Meier plot and cox model results based on a predicted probability threshold (>30%) from the multivariable prediction models (i.e., time from sleep study to first severe exacerbation)**

| **Adjusted prediction model** | **Simple Adjusted model with less predictors** |
| --- | --- |
| 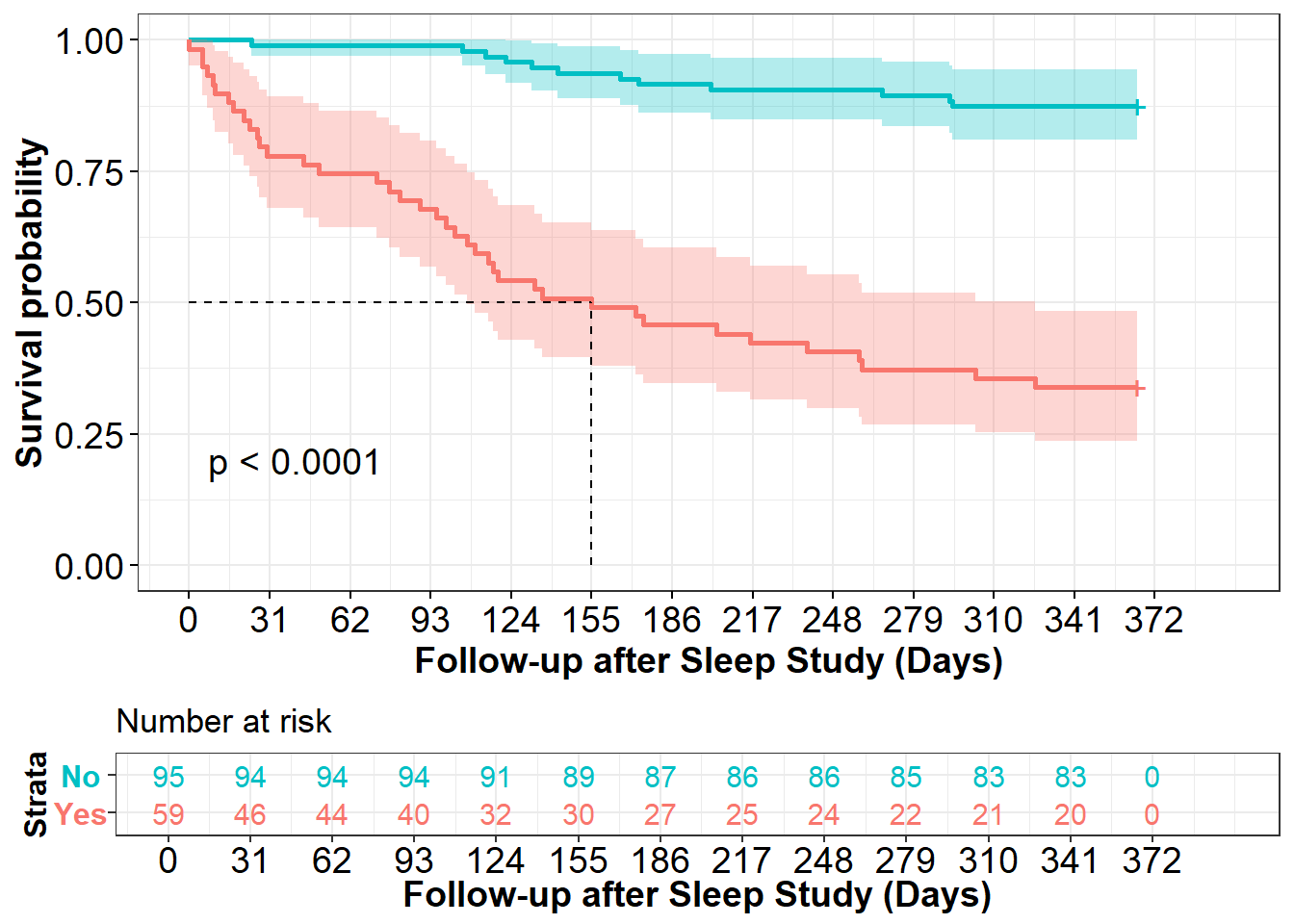  High vs Low: HR: 8.37; 95%CI: 4.36, 16.01 | 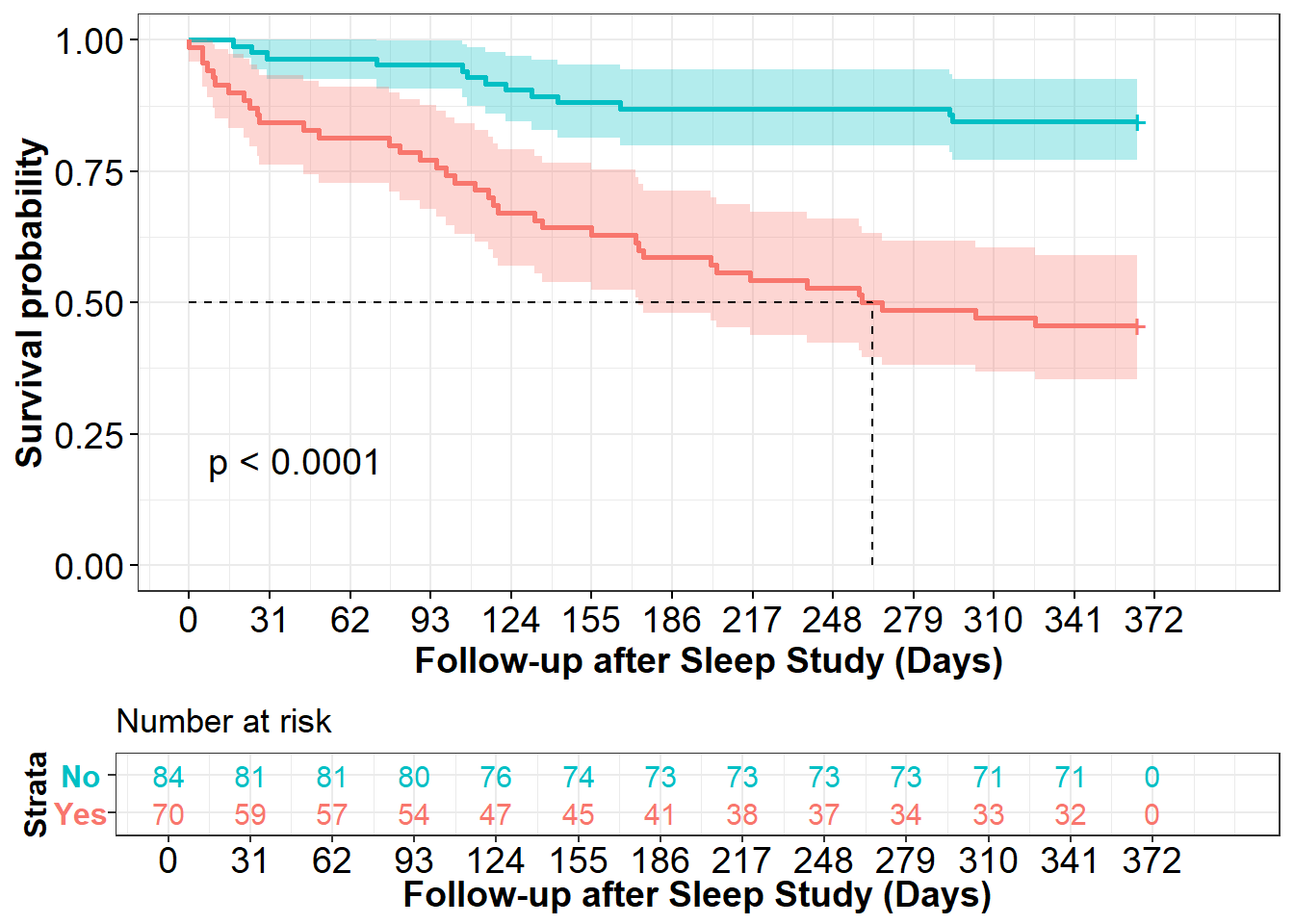  High vs Low: HR: 4.60; 95%CI: 2.44, 8.64 |

**Supplementary Figure 10.** Time-varying Receiver Operating Curve Analysis of the Adjusted and Crude Models


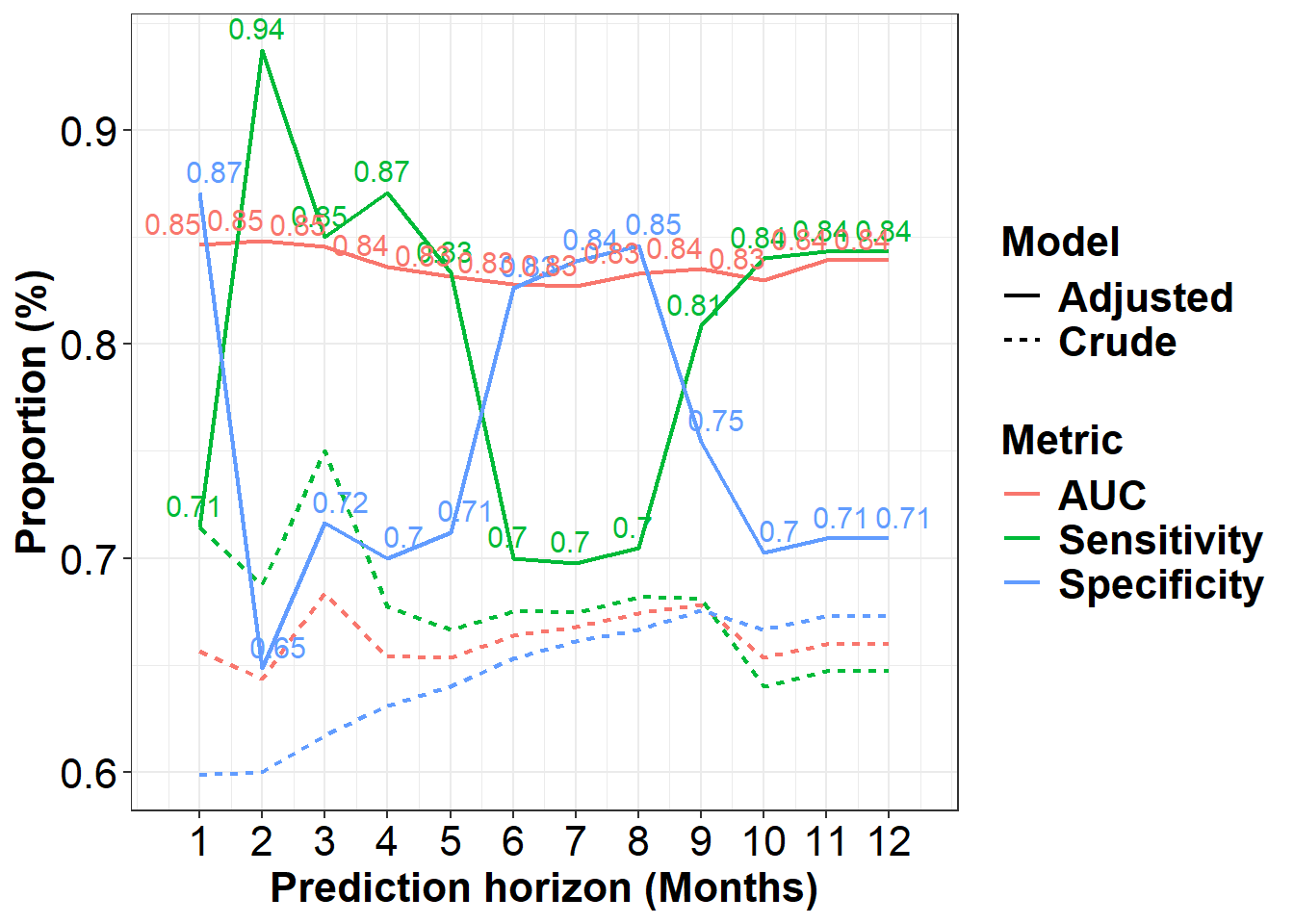


**Supplemental Table 1. Definitions and descriptions of sleep related measures**

| **Measure** | **Description** |
| --- | --- |
| Total REM sleep time spent with CO2 above 50 mm Hg: | Total time in REM spent with CO2 > 50 mm Hg (minutes)x100  Stage REM sleep |
| Percentage of sleep time spent in light stages of sleep: | Stage 1 sleep +Stage 2 sleep  Total sleep time |
| Percentage of sleep time spent in deep stage of sleep: | Stage 3 sleep  Total sleep time |
| Percentage of sleep time spent in REM stage of sleep: | Stage REM sleep  Total sleep time |
| Percentage of Time Spent in Bed below 90% OSat | Total Time in Bed Spent Below 90% (min) x100  Total sleep time |

Electroencephalogram was collected by placement of frontal [F3,F4], central [C3,C4], occipital [O1, O2] referenced to the opposite mastoid electrodes [M1,M2]) and utilized to score stage 1,2, 3 and REM sleep. Electro-oculogram, electromyogram (chin and both legs), electrocardiogram, pressure transducer and thermistor airflow, uncalibrated respiratory inductance plethysmography, oximetry, and end-tidal CO_2_ (ETCO_2_) data with video monitoring of the study for scoring support was collected. Pressure transducer and thermistor airflow, uncalibrated respiratory inductance plethysmography were utilized to score respiratory events and assisted in Apnea hypopnea index (AHI) calculation. PSG variable data were collected using the following equipment: Alice G3 PSG software (Pleasanton, California, United States), nasal airflow and (Dynamedix Diagnostics, Shoreview, Minnesota, United States; Salter Labs, Arvin, California, United States), Protech 2 channel pediatric thermistor (Philips Respironics, Murrysville, Pennsylvania, United States), oximetry (Nonin for internal Plymouth, MN, United States and Massimo for external, Irvine, California, United States), respiratory inductance plethysmography (PerfectFit, Dynamedix Diagnostics, Shoreview, Minnesota, United States), and Capnography (Respironics LOFLO, Murrysville, Pennsylvania, United States and Sentec for TCM, Lincoln, RI, United States).

**Supplemental Table 2. Definitions and descriptions of early childhood asthma risk factors**

| **Key Terms** | **Early childhood predictors of asthma burden** | **ICD Codes** |
| --- | --- | --- |
| Asthma (Outcome) | Physician documented asthma diagnosis based on icd9/10 code/text | J45.xx, 493.xx |
| Pre-school age asthma (PF) | Physician documented asthma diagnosis based on icd9/10 code/text at ages ≤3 years | J45.xx, 493.xx |
| Black or African American Race | Documented self-report |  |
| Parental History of Asthma | Documented self-report | Z82.5, V17.5 |
| Wheezing | Physician documented wheeze symptoms or diagnosis in the first 3 years of life | 786.07, J80, R09.2, J96.xx, 518.82, 518.81, 799.1, R06.2 |
| Wheezing without Cold | Physician documented wheeze symptoms /diagnosis that **did not** include cold related symptoms/diagnosis in the first 3 years of life | ICD codes used for colds: J00, J31.1, J06.0, J06.9, 465.9, 460 |
| Eczema | Physician documented diagnosis of Eczema in the first 3 years of life | 692.5, 692.6, L30.x, L20 |
| Polysensitization/  Multiple allergies^1^ | Two or more physician documented allergy diagnosis including allergy related reactions related to aeroallergens or foods  OR  Two or more laboratory results of positive allergy sensitization related to aeroallergens or foods based on skin prick test identified as an allergen wheal was ≥3 mm greater than the saline control or a positive allergy blood test of specific immunoglobulin E (IgE) ≥0.35 kU/l.  OR  At least one allergy diagnosis and one positive allergy sensitization result related to aeroallergens or foods | Z91.0, 995.3, T78.40XA |
| Pneumonia | Physician documented of a pneumonia diagnosis in the first three years of life | 481, 482, 483, 484, 485, 486, 487.0, J12, J13, J14, J15, J16, J17, J18 |
| Bronchiolitis | Physician documented a bronchiolitis diagnosis in the first 3 years of life | 466.1, 466.11, 466.19, J21.0, J21.1, J21.8, J21.9 |

^1^Descriptors of allergies/polysensitization documented in the EHR: (1) Pollen allergies include allergy to elm, pollen; (2) Dust/Dust Mites allergies include allergy to dust, dust mites, and dander; (3) Nut allergies include allergy to almonds, cashews, pistachios, pecans, peanuts, tree nuts, walnuts, and nuts; (4) Egg allergies include allergy to eggs; (5) Seafood allergies include allergy to crab, lobster, shrimp, fish, shellfish, swordfish, tuna, scallops, and seafood; (6) Seasonal allergies include allergy to fall, winter, autumn, summer, and seasonal; (7) Milk allergies include allergy to milk, and dairy; (8) Fruit allergies include allergy to banana, coconut, pineapple, kiwi, raspberry, strawberry, watermelon, mango and fruits; (9) Pet allergies include allergy to cats, dogs, mice, rodents, pet dander, animal dander, and animals; (10) Drug allergies include allergy to ibuprofen, amoxicillin, Augmentin, Tylenol, erythromycin, antibiotics, penicillin, propylene glycol and drugs; (11) Grass allergies include allergy to grass, and hay; (12) Insects allergies include allergy to wasps, cockroaches, insect stings, bees, hornets and insects; (13) Smoke allergies include allergy to smoke; (14) Mold allergies include allergy to mold; (15) Fur allergies include allergy to fur; (16) Trees/Plants allergies include allergy to birch, oak, pigweed, ragweed, trees and plants; (17) Other-Food allergies include allergy to wheat, gluten, rice, pork, soy, sunflower seeds and foods; (18) Other-General allergies include allergy to adhesive bandages, adhesives, dyes, environment, latex, metals, narcotics, perfumes, and other.

**Supplementary Table 3. Distribution and association between sleep measures and risk of severe exacerbations (SE) post-sleep study**

| Characteristic | Overall | No SE | SE | p-value | Odds Ratios (95%CI) | |
| --- | --- | --- | --- | --- | --- | --- |
|  | N = 161 | N = 109 | N = 52 |  | **Crude** | **Adjusted^1^** |
| CO2 Estimation: Median (Q1, Q3) | 2 (2, 2) | 2 (2, 2) | 2 (2, 2) | 0.416 | 1.25 (0.38, 4.87) | 0.70 (0.19, 2.96) |
| OAHI: Median (Q1, Q3) | 0 (0, 1) | 0 (0, 1) | 0 (0, 1) | 0.914 | 0.97 (0.86, 1.03) | 0.99 (0.88, 1.06) |
| Central apnea index:  Median (Q1, Q3) | 0 (0, 1) | 0 (0, 1) | 0 (0, 1) | 0.245 | 0.93 (0.74, 1.08) | 0.92 (0.72, 1.09) |
| Stage 1 Sleep:  Mean (SD) | 34 (25) | 33 (24) | 34 (29) | 0.667 | 1.00 (0.99, 1.01) | 1.01 (0.99, 1.02) |
| Stage 2 Sleep:  Mean (SD) | 183 (60) | 177 (60) | 194 (58) | 0.063 | 1.00 (1.00, 1.01) | 1.01 (1.00, 1.01) |
| Stage 3 Sleep:  Mean (SD) | 114 (44) | 115 (45) | 111 (43) | 0.350 | 1.00 (0.99, 1.01) | 0.99 (0.98, 1.00) |
| Stage REM Sleep:  Mean (SD) | 63 (32) | 61 (34) | 67 (28) | 0.289 | 1.01 (1.00, 1.02) | 1.01 (0.99, 1.02) |
| Total Sleep Time:  Mean (SD) | 393 (76) | 387 (77) | 406 (71) | 0.083 | 1.00 (1.00, 1.01) | 1.00 (1.00, 1.01) |
| Total REM sleep time spent with CO2 > 50 mm Hg (min):  Median (Q1, Q3) | 0 (0, 1) | 0 (0, 1) | 0 (0, 1) | 0.794 | 1.01 (0.98, 1.04) | 1.01 (0.98, 1.04) |
| Total NREM sleep time spent with CO2 > 50 mm Hg (min):  Median (Q1, Q3) | 0 (0, 9) | 0 (0, 9) | 0 (0, 4) | 0.681 | 1.00 (1.00, 1.01) | 1.00 (1.00, 1.01) |
| Total (%) REM Sleep Time Spent with CO2 > 50 mm Hg:  Median (Q1, Q3) | 0 (0, 4) | 0 (0, 4) | 0 (0, 4) | 0.583 | 1.01 (0.99, 1.02) | 1.01 (0.99, 1.03) |
| Total (%) NREM Sleep Time Spent with CO2 > 50 mm Hg:  Median (Q1, Q3) | 0 (0, 2) | 0 (0, 3) | 0 (0, 1) | 0.664 | 1.01 (1.00, 1.02) | 1.01 (0.98, 1.03) |
| Total Time in Bed Spent Below 90% (min): Median (Q1, Q3) | 0 (0, 2) | 0 (0, 2) | 0 (0, 2) | 0.624 | 1.00 (0.96, 1.04) | 1.00 (0.96, 1.05) |
| Sleep Efficiency: Median (Q1, Q3) | 80 (70, 89) | 79 (69, 88) | 86 (76, 91) | **0.025** | **1.03 (1.00, 1.06)** | **1.03 (1.00, 1.06)** |
| Average Saturations:  Mean (SD) | 96 (1) | 96 (1) | 96 (1) | 0.205 | 0.86 (0.68, 1.09) | 0.82 (0.63, 1.05) |
| AHI:  Median (Q1, Q3) | 7 (3, 14) | 7 (3, 15) | 7 (4, 14) | 0.810 | 1.00 (0.98, 1.02) | 1.01 (0.98, 1.03) |
| OAHI |  |  |  | 0.505 |  |  |
| Normal (<5), % | 150 (93.2) | 100 (91.7) | 50 (96.2) |  | — | — |
| Pathological (>= 5), % | 11 (6.8) | 9 (8.3) | 2 (3.8) |  | 0.41 (0.06, 1.68) | 0.50 (0.07, 2.33) |
| Central apnea index |  |  |  | 0.417 |  |  |
| Normal (<1), % | 121 (75.2) | 84 (77.1) | 37 (71.2) |  | — | — |
| Pathological (>= 1), % | 40 (24.8) | 25 (22.9) | 15 (28.8) |  | 1.32 (0.61, 2.79) | 1.24 (0.54, 2.78) |
| CO2 while asleep: Mean (SD) | 43 (4) | 43 (4) | 43 (4) | 0.575 | 0.98 (0.89, 1.08) | 0.97 (0.86, 1.09) |
| Light Stage of Sleep: % Median (Q1, Q3) | 54 (47, 63) | 52 (45, 63) | 57 (49, 63) | 0.281 | 1.01 (0.98, 1.03) | 1.02 (0.99, 1.06) |
| Deep Stage of Sleep: % Median (Q1, Q3) | 30 (23, 37) | 30 (23, 37) | 28 (20, 35) | 0.137 | 0.98 (0.95, 1.01) | 0.97 (0.93, 1.00) |
| REM Stage of Sleep: % Median (Q1, Q3) | 16 (11, 20) | 16 (11, 20) | 16 (12, 20) | 0.473 | 1.03 (0.98, 1.08) | 1.02 (0.96, 1.08) |
| Average saturations in REM: Mean (SD) | 96 (2) | 96 (2) | 96 (2) | 0.657 | 0.98 (0.83, 1.17) | 0.95 (0.79, 1.15) |
| Nadir saturation: Median (Q1, Q3) | 88 (83, 91) | 88 (83, 91) | 87 (84, 90) | 0.383 | 1.00 (0.94, 1.05) | 0.99 (0.93, 1.05) |

^1^ Adjusted ORs control for age at sleep study, age of asthma diagnosis, sex, race/ethnicity, history of severe exacerbations.

**Supplementary Table 4. Distribution and association between of demographic factors, medication history, and risk of severe exacerbations (SE) 12-months post-sleep study**

| **Patient Characteristics** | **Overall** | **No SE** | **SE** | **p value** | **Odds Ratio (95%CI)** | |
| --- | --- | --- | --- | --- | --- | --- |
| **Overall** | **161** | 101 (67.7%) | 52 (32.3%) |  | **Crude** | **Adjusted^1^** |
| **BMI at Sleep Study** |  |  |  | 0.337 | 1.00 (0.97, 1.03) | 1.02 (0.97, 1.06) |
| Mean (SD) | 33 (10) | 33 (11) | 33 (10) |  |  |  |
| Median (Q1, Q3) | 32 (26, 38) | 31 (26, 38) | 32 (26, 39) |  |  |  |
| **ACT Score 1-year before PSG Date** |  |  |  | 0.834 | 1.02 (0.97, 1.06) | 1.02 (0.97, 1.08) |
| Mean (SD) | 22 (8) | 22 (6) | 23 (11) |  |  |  |
| Median (Q1, Q3) | 21 (19, 23) | 21 (19, 23) | 21 (18, 24) |  |  |  |
| **FEV1 Percent Predicted at PSG Date** |  |  |  | 0.609 | 0.99 (0.96, 1.02) | 0.98 (0.95, 1.02) |
| Mean (SD) | 102 (12) | 103 (12) | 101 (11) |  |  |  |
| Median (Q1, Q3) | 103 (96, 108) | 103 (97, 108) | 103 (95, 108) |  |  |  |
| **Allergy ≤ 3 yrs** |  |  |  | 0.302 |  |  |
| No | 96 (59.6%) | 68 (70.8%) | 28 (29.2%) |  | — | — |
| Yes | 65 (40.4%) | 41 (63.1%) | 24 (36.9%) |  | 1.38 (0.70, 2.73) | 1.18 (0.56, 2.47) |
| **Eczema ≤ 3 yrs** |  |  |  | 0.303 |  |  |
| No | 99 (61.5%) | 70 (70.7%) | 29 (29.3%) |  | — | — |
| Yes | 62 (38.5%) | 39 (62.9%) | 23 (37.1%) |  | 1.52 (0.76, 3.01) | 1.36 (0.63, 2.92) |
| **Parental Asthma** |  |  |  | 0.925 |  |  |
| No | 120 (74.5%) | 81 (67.5%) | 39 (32.5%) |  | — | — |
| Yes | 41 (25.5%) | 28 (68.3%) | 13 (31.7%) |  | 0.93 (0.42, 1.97) | 0.89 (0.38, 2.00) |
| **Wheezing ≤ 3 yrs** |  |  |  | **0.030** |  |  |
| No | 94 (58.4%) | 70 (74.5%) | 24 (25.5%) |  | — | — |
| Yes | 67 (41.6%) | 39 (58.2%) | 28 (41.8%) |  | 1.96 (1.00, 3.89) | 1.65 (0.79, 3.45) |
| **Wheezing w/o Cold ≤ 3 yrs** |  |  |  | **0.030** |  |  |
| No | 94 (58.4%) | 70 (74.5%) | 24 (25.5%) |  | — | — |
| Yes | 67 (41.6%) | 39 (58.2%) | 28 (41.8%) |  | 1.96 (1.00, 3.89) | 1.65 (0.79, 3.45) |
| **Bronchiolitis ≤ 3 yrs** |  |  |  | **0.020** |  |  |
| No | 118 (73.3%) | 86 (72.9%) | 32 (27.1%) |  | — | — |
| Yes | 43 (26.7%) | 23 (53.5%) | 20 (46.5%) |  | 2.27 (1.09, 4.74) | 1.97 (0.87, 4.53) |
| **Pneumonia ≤ 3 yrs** |  |  |  | 0.402 |  |  |
| No | 82 (50.9%) | 58 (70.7%) | 24 (29.3%) |  | — | — |
| Yes | 79 (49.1%) | 51 (64.6%) | 28 (35.4%) |  | 1.26 (0.65, 2.48) | 0.80 (0.37, 1.69) |
| **Medication History** |  |  |  |  |  |  |
| **ICS One Year Before PSG** |  |  |  | >0.999 |  |  |
| No | 10 (6.2%) | 7 (70.0%) | 3 (30.0%) |  | — | — |
| Yes | 151 (93.8%) | 102 (67.5%) | 49 (32.5%) |  | 1.20 (0.32, 5.77) | 0.49 (0.12, 2.53) |
| **ICS plus LABA** **One Year Before PSG** |  |  |  | **<0.001** |  |  |
| No | 65 (40.4%) | 71 (82.6%) | 15 (17.4%) |  | — | — |
| Yes | 96 (59.6%) | 38 (50.7%) | 37 (49.3%) |  | 4.52 (2.23, 9.55) | 2.60 (0.97, 6.92) |
| **Hydroxyzine One Year Before PSG** |  |  |  | **0.032** |  |  |
| No | 152 (94.4%) | 100 (65.8%) | 52 (34.2%) |  | — | — |
| Yes | 9 (5.6%) | 9 (100.0%) | 0 (0.0%) |  | - | - |
| **Melatonin One Year Before PSG** |  |  |  | 0.456 |  |  |
| No | 141 (87.6%) | 94 (66.7%) | 47 (33.3%) |  | — | — |
| Yes | 20 (12.4%) | 15 (75.0%) | 5 (25.0%) |  | 0.62 (0.19, 1.70) | 0.64 (0.18, 1.98) |
| **Clonidine One Year Before PSG** |  |  |  | >0.999 |  |  |
| No | 152 (94.4%) | 103 (67.8%) | 49 (32.2%) |  | — | — |
| Yes | 9 (5.6%) | 6 (66.7%) | 3 (33.3%) |  | 0.98 (0.20, 3.88) | 0.86 (0.15, 3.99) |
| **Trazodone One Year Before PSG** |  |  |  | >0.999 |  |  |
| No | 157 (97.5%) | 106 (67.5%) | 51 (32.5%) |  | — | — |
| Yes | 4 (2.5%) | 3 (75.0%) | 1 (25.0%) |  | 0.65 (0.03, 5.20) | 1.34 (0.06, 12.4) |

^1^ Adjusted ORs control for age at sleep study, age of asthma diagnosis, sex, race/ethnicity, history of severe exacerbations.
